# Network meta-analysis combining individual patient and aggregate data from a mixture of study designs with an application to pulmonary arterial hypertension

**DOI:** 10.1186/s12874-015-0007-0

**Published:** 2015-04-12

**Authors:** Howard HZ Thom, Gorana Capkun, Annamaria Cerulli, Richard M Nixon, Luke S Howard

**Affiliations:** School of Social and Community Medicine, Bristol, UK; Novartis Pharma AG, Basel, Switzerland; National Heart & Lung Institute, Imperial College London, London, UK

**Keywords:** Network meta-analysis, Individual patient data, Covariate adjustments, Observational evidence, Mixed treatment comparison, Pulmonary arterial hypertension

## Abstract

**Background:**

Network meta-analysis (NMA) is a methodology for indirectly comparing, and strengthening direct comparisons of two or more treatments for the management of disease by combining evidence from multiple studies. It is sometimes not possible to perform treatment comparisons as evidence networks restricted to randomized controlled trials (RCTs) may be disconnected. We propose a Bayesian NMA model that allows to include single-arm, before-and-after, observational studies to complete these disconnected networks. We illustrate the method with an indirect comparison of treatments for pulmonary arterial hypertension (PAH).

**Methods:**

Our method uses a random effects model for placebo improvements to include single-arm observational studies into a general NMA. Building on recent research for binary outcomes, we develop a covariate-adjusted continuous-outcome NMA model that combines individual patient data (IPD) and aggregate data from two-arm RCTs with the single-arm observational studies. We apply this model to a complex comparison of therapies for PAH combining IPD from a phase-III RCT of imatinib as add-on therapy for PAH and aggregate data from RCTs and single-arm observational studies, both identified by a systematic review.

**Results:**

Through the inclusion of observational studies, our method allowed the comparison of imatinib as add-on therapy for PAH with other treatments. This comparison had not been previously possible due to the limited RCT evidence available. However, the credible intervals of our posterior estimates were wide so the overall results were inconclusive. The comparison should be treated as exploratory and should not be used to guide clinical practice.

**Conclusions:**

Our method for the inclusion of single-arm observational studies allows the performance of indirect comparisons that had previously not been possible due to incomplete networks composed solely of available RCTs. We also built on many recent innovations to enable researchers to use both aggregate data and IPD. This method could be used in similar situations where treatment comparisons have not been possible due to restrictions to RCT evidence and where a mixture of aggregate data and IPD are available.

**Electronic supplementary material:**

The online version of this article (doi:10.1186/s12874-015-0007-0) contains supplementary material, which is available to authorized users.

## Background

Decision making bodies for national health care providers, such as the National Institute for Health and Care Excellence (NICE) for the NHS in England and Wales or the Pharmaceutical Benefits Advisory Committee (PBAC) in Australia, have a need to consider all available treatments when making recommendations for clinical practice. There is rarely a single definitive study comparing these treatments and it is often necessary to synthesise the best available evidence to come to a decision [1].

Network meta-analysis (NMA) for indirect mixed treatment comparisons of multiple treatments is a generalization of standard meta-analysis, which is used to combine the results of multiple studies, to the comparison of two or more than treatments. This has become a well-established methodology for evidence synthesis [2,3] and is routinely used and recommended by NICE [4,5]. The gold-standard of evidence to be included in a NMA are randomized controlled trials (RCTs) which include a control arm and whose populations are randomized to reduce bias and improve precision. The results are usually available from literature as only aggregate data. Access to individual patient data (IPD), when available, can be used to understand the relationship between covariates and outcomes [6,7]. Methods for the inclusion of IPD in pairwise meta-analysis have been developed by Sutton *et al.* [8] and Riley *et al.* [9,10] and these were extended to the network meta-analysis of binary outcomes by Saramago et al. [7] and Donegan et al. [6]. This model can easily be adapted to continuous outcomes and provides a covariate-adjusted NMA model combining IPD and aggregate data.

**Figure 1:**
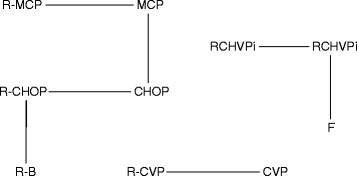
Example of a disconnected network from network meta-analysis of first-line treatments for stage III-IV follicular lymphoma.

One of the requirements to perform an NMA is to have a connected network [4], which can be challenging when not enough RTCs are available, as illustrated in Figure 1 for the case of a NICE technology assessment follicular lymphoma [11]. This is often a problem in new indications for small populations or orphan diseases [12]. However, a decision on the most appropriate treatment is still needed and including non-randomized studies to complete the network and conduct the comparison is a potential solution [13]. A commonly available type of non-randomized study is the single-arm observational study, or before-and-after study [14], in which outcomes in a group of patients are investigated before and after an intervention.

Several methods have been proposed to incorporate such observational studies [15]. One approach is the three-level hierarchical model which allows the incorporation of evidence from many different study designs [16,17]. An example of such a model consists of an overall effect for each treatment *j*, which can be labelled *d*_*j*_. Treatment effects for each different type of study, such as an RCT effect *φ*_*j*1_, a before-and-after study effect *φ*_*j*2_, and a case-control study effect *φ*_*j*3_, could then be normally distributed around this overall effect. At the bottom level of the hierarchy are the individual study effects *δ*_*jki*_ for each treatment *j*, study type k, and study *i*, which could be normally distributed around the study type treatment effects *φ*_*jk*_. This approach has the advantage of keeping the inference from each type of trial separate but is not applicable in cases where the number of studies per study type per treatment is small.

An alternative approach to including observational studies, and thus connecting the network, is that of propensity scores which are the probability that a patient would be given a particular treatment on the basis of their background characteristics [18-20]. These probabilities are often estimated using logistic regression. However, different propensity score models are required for each treatment and a great many studies are therefore required for each study. This is a particular drawback if IPD is not available for most of the treatments. Another disadvantage is the difficulty of incorporating propensity scores into the existing covariate-adjusted NMA models.

A final alternative for including observational studies in disconnected networks is the method of constructing empirical priors informed by these observational studies [15,21]. These empirical priors inform parameter estimation via:

$$ P\left(\left.\theta \right| Data\right)\alpha L\left(\left.\theta \right| RCTs\right)\times {\left[L\left(\left.\theta \right|Obs\right)\right]}^{\alpha }P\left(\theta \right) $$

where the *L*(*θ*|*RCTs*) is the likelihood on the basis of the RCT evidence, *L*(*θ*|*Obs*) is the likelihood on the basis of the observational evidence, *P*(*θ*) is the prior, and *α* is a parameter representing the strength given to the observational evidence. If *α* = 1, for example, the observational evidence would be given the same weight as the RCT evidence. This approach shares the advantage of the hierarchical method in that it explicitly separates the RCT and observational evidence but also shares the disadvantage of the propensity scores method that it is difficult to merge with existing NMA models.

The method we choose to build upon is the construction of control arms for before-and-after studies by matching their baseline characteristics to those of control arms in included RCTs [20,22]. This analysis of covariance method uses regression models to estimate the effect of treatments not included in the study. We adapted this in a natural fashion to covariate-adjusted NMA models through an assumption of exchangeability (random-effects) on the placebo effects of study arms. A similar approach was originally applied to meta-analysis [23] and has recently been proposed for the construction of baseline natural history models in NMA [24]. However, recent work has been critical of placing random-effects on the trial-level baseline improvements, namely the placebo effect [24,25], as it interferes with the randomization of the RCTs and learns across trial information. Despite these concerns, in cases such as the application we will discuss necessitate this approach as it would otherwise not be possible to compare the treatments of interest due to the disconnectedness of the evidence network.

### Illustrative example: mixed treatment comparison of combination therapies for pulmonary arterial hypertension

We will illustrate our method for the inclusion of before-and-after studies in a mixed treatment comparisons of therapies for pulmonary arterial hypertension (PAH). PAH is a rare disease characterised by progressive elevation of pulmonary vascular resistance leading to right heart failure and death [26]. Current treatments include endothelin receptor antagonists (ERA), phosphodiesterase-5 inhibitors (PDE5i), and prostacyclin analogues (Pr) [27]. These drugs are often used in combination to try to improve outcomes [28,29]. The anticancer therapy imatinib is an oral therapy which has also recently been studied in PAH and its use is of interest to clinicians. No systematic comparison of available monotherapies and combination therapies for PAH has been conducted and, in particular, imatinib as add-on to other combination therapies has not been investigated. Imatinib was being evaluated as an alternative to prostacyclins as additional therapy for patients on a combination of ERA and PDE5i. The comparison of imatinib with prostacyclins for this patient group was not previously possible on the basis of direct evidence or through indirect NMA comparisons restricted to RCTs and we thus took it as our primary objective for treatment comparison. However, our comparison should be viewed as illustrative and should not be used to guide clinical practice as the evidence is indirect and the analysis relies on a number of model assumptions that were necessary to facilitate the comparison.

Our primary evidence base for the NMA was the IPD from the IMPRES trial [30]. This was a randomized placebo-controlled Phase-III trial to investigate the efficacy and safety of imatinib as an add-on to combination therapy for the treatment of PAH. The study included patients with severe PAH and receiving two or more PAH-specific treatments^.^. Patients were initially on one of four combination treatments, namely ERA + PDE5i, ERA + Pr, PDE5i + Pr, or ERA + PDE5i + Pr., were randomized within these background treatment groups to either imatinib or placebo and were followed-up for at least 24 weeks. Patient group characteristics are reported in Table 1, where heterogeneity in baseline characteristics between randomized groups is exhibited. The high dropout rates in this trial reflect the severity of the disease and the side-effects of the treatments.

**Table 1:** **Summary statistics of patients in IMPRES RCT**

**Baseline therapy**	**Add-on Therapy**	**Size**¶	**Drop out§**	**Mean 6MWD improvement**	**Mean age**	**Prop male**	**Mean STATUS**	**Mean 6MWD baseline**	**Mean PVR**
ERA + PDE5i	Placebo*	23	4	2.54 (16.25)	50.30	0.30	2.96	342.07	1157.0
ERA + PDE5i	Imatinib	20	12	43.7 (14.27)	50.15	0.10	2.60	328.95	1282.1
ERA + Pr	Placebo	8	2	7.81 (14.36)	37.00	0.25	2.38	381.56	1146.6
ERA + Pr	Imatinib	10	5	48.3 (16.12)	47.20	0.00	2.80	331.60	1071.9
ERA + PDE5i + Pr	Placebo	33	8	−8.27 (10.47)	47.03	0.18	2.73	355.79	1176.6
ERA + PDE5i + Pr	Imatinib	27	15	33.37 (11.54)	47.74	0.19	2.85	360.70	1232.5
PDE5i + Pr	Placebo	16	4	36.03 (10.53)	43.19	0.06	2.56	358.72	1193.9
PDE5i + Pr	Imatinib	9	5	40 (14.59)	53.00	0.11	2.78	380.56	1050.8

*ERA is any endothelin receptor antagonist, PDE5i is phosphodiesterase 5 inhibitor, and Pr is prostacyclins (oral, inhaled, intravenous or subcutaneous).

¶ Group size was number of patients taking 6MWD test at baseline and 24 weeks.

§ Dropout is number of patients dropping out of the study between baseline and 24 weeks. Dropout due to death, adverse events, consent withdrawal, protocol deviation, abnormal laboratory result, administrative error, or adverse reaction to study drug.

The continuous outcome of short term change in 6 minute walk distance (6MWD) from baseline, in meters, is used in licensing decisions by agencies such as the Food and Drug Administration [31] and as a result is the primary outcome in nearly all Phase-III trials in PAH. Although adjusting final 6MWD for baseline 6MWD is the recommended approach when analysing trial results [32], this was not possible as we had only aggregate data from most of the studies and many only reported the change outcome. We therefore chose change in 6MWD as our efficacy measure. Short term was defined as 12 weeks to 1 year, as clinical opinion was that patients would derive maximal benefit from treatments within 12 weeks.

Six covariates of interest were identified by a mixture of exploratory analysis of the IMPRES data and expert clinical opinion. The covariates identified were: the age at baseline (AGE), an indicator for whether a patient is male (SEX), the 6MWD at baseline (WALK), the World Health Organization New York Health Assessment status (STATUS) and pulmonary vascular resistance (PVR). STATUS categorises the severity of PAH into one of four increasingly severe categories, ranging from no limitation of activity and no symptoms with ordinary physical activity to marked limitation of activity and symptoms with any activity, even at rest. PVR is a measure of the resistance of the pulmonary vasculature calculated from the pressure drop across the pulmonary vascular bed divided by the pulmonary blood flow. Means of the covariates were used for the aggregate data.

### Systematic literature review of studies in the literature

**Figure 2:**
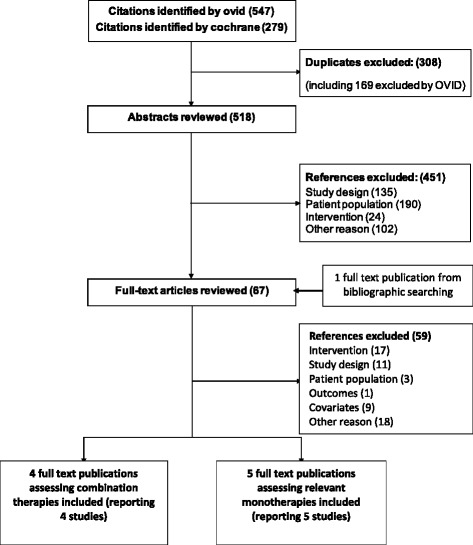
PRISMA flowchart for selection of monotherapy and combination therapy RCTs.

**Figure 3:**
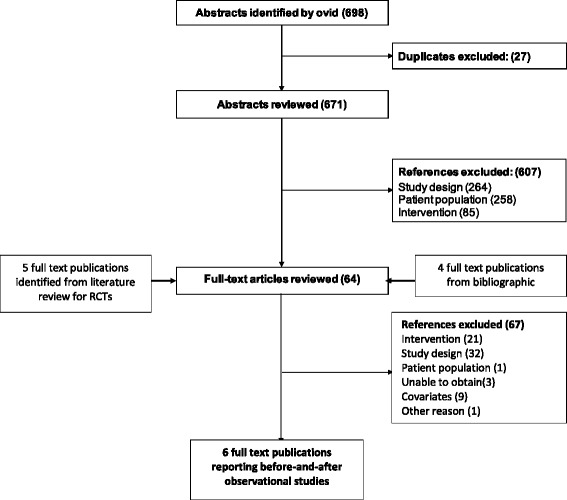
PRISMA flowchart for selection of monotherapy and combination therapy observational studies.

The results of a systematic literature review were available and was used to identify a network of studies to be included in the analysis. In this review, the MEDLINE® and EMBASE® databases were searched simultaneously. Patient Intervention Comparator Outcome Study type (PICOS) [33] criteria were followed and the quality assessment was performed according to the NICE checklist for RCTs [34]. Details of the PICOS terms are included in Additional file 1. Search terms included a combination of free-text and thesaurus terms relevant to PAH, ERA, prostacyclins, PDE5i, and RCTs, although case-control and cohort studies were also included. The Cochrane Central Register of Controlled Trials was also searched using a similar strategy. The relevance of each citation identified from the databases was based on title and abstract according to the PICOS criteria. As we wanted to explore the effects of covariates, studies that did not report two or more of the 6 covariates of interest were excluded, while we would use IMPRES data to perform single imputation when only one covariate is missing. From this review, we identified and included 5 monotherapy [35-39] and 4 combination therapy [28,40-42] RCTs, summary statistics for which are provided in Table 2 and Table 3, respectively. Additionally, 6 before-and-after studies investigating monotherapies and combination therapies were included [43-48] and their summary statistics are reported in Table 4. PRISMA flowcharts are provided for the systematic searches in Figure 2 and Figure 3 and a PRISMA checklist is provided in Additional file 2 [49]. Although there were substantial differences across trials in the doses of the administered treatments, as recorded in Tables 2, 3 and 4, clinical opinion was such that their effects would be comparable.

**Table 2:** Details of included monotherapy RCTs*

**Reference**	**Badesch 2002**		**Rubin 2002 (BREATHE-1)**		**Barst 2006 (STRIDE-2)**		**Barst 1996**		**Galie 2005 (SUPER-1)**	
**Baseline therapy**	None		None		None		Conventional		None	
**Add-on therapy**	ERA	Placebo	ERA	Placebo	ERA	Placebo	Pr (iv ep)	Conventional	PDE5i	Placebo
**Treatment dose**	62.5 mg bosentan twice daily, increased to 125 mg twice daily after 4 weeks.		62.5 mg bosentan twice daily, increased to either 125 mg or 250 mg twice daily after 4 weeks.		62.5 mg bosentan twice daily, increased to 125 mg twice daily after 4 weeks.		mean dose of intravenous epoprostenol 9.2 ng/kg/min		80 mg sildenafil orally 3 times daily	
**Patients at end of trial**	21	11	144	69	60	62	41	40	71	70
**Duration**	12 weeks		16 weeks		18 weeks		12 weeks		12 weeks	
**Change 6MWD**	70 (23.4)	−6 (50.5)	36 (6.5)	−8 (9.5)	23 (9.3)	−6.5 (9.2)	32 (24.8)	−15 (33)	50 (9)	2 (7)
**Baseline 6MWD**	360 (18.8)	355 (24.7)	330 (6.2)	344 (9.1)	337 (10.1)	321 (10.8)	316 (18)	272 (23.0)	339 (9.4)	344 (9.4)
**Age**	52.2	47.4	48.7	47.2	49	53	40	40	48	49
**Sex (% male)**	0.19	0	0.21	0.22	0.22	0.24	0.24	0.3	0.21	0.19
**STATUS**	3	3	3.097222	3.057971	2.65	2.693548	3.243902	3.275	2.619718	2.557143
**PVR**	896	942	1014	880	880	880	1280	1280	918	1051

*ERA are endothelin receptor antagonists, PDE5i are phosphodiesterase 5 inhibitors, Pr are prostacyclin analogues. iv ep is intravenous epoprostenol.

**Table 3:** Details of included combination therapy RCTs*

**Reference**	**Barst 2011 (PHIRST-1)**		**Simonneau 2008 (PACES)**		**McLaughlin 2006 (STEP)**		**Humbert 2004 (BREATHE-2)**	
**Baseline therapy**	ERA		Pr (iv epo)		ERA		None	
**Add-on therapy**	PDE5i	Placebo	PDE5i	Placebo	Pr (inh ilp)	Placebo	ERA	ERA+ Pr (iv epo)
**Treatment dose**	40 mg tadalafil once daily		3x20mg sildenafil daily, increased to 40 and 80 at 4 week intervals		5 μg inhaled iloprost		62.5 mg bosentan twice daily, increased to 125 mg twice daily after 4 weeks. Intravenous epoprostenol started at 2 ng/kg/min and increased up to 14 ± 2 ng/kg/min after 16 weeks.	
**Patients at end of trial**	42	45	133	123	34	33	19	10
**Duration**	16 weeks		16 weeks		12 weeks		16 weeks	
**Change 6MWD**	40.2 (8.5)	18.8 (9.2)	29.8 (5.3)	1 (5.3)	30 (10.3)	4 (10.6)	72 (11.47)	46 (19.61)
**Baseline 6MWD**	360.9 (11.6)	348.5 (12.7)	348.9 (6.2)	341 (6.7)	331 (73)	340 (64)	323.04§	323.62§
**Age**	50	51.7	47.8	47.5	49	51	45	47
**Sex (% male)**	0.21	0.22	0.18	0.23	0.21	0.21	0.23	0.45
**STATUS**	2.5	2.7	2.8	2.8	3.0	3.0	3.23	3.27
**PVR**	863.3	863.3	856.8	754.9	815	783	1511	1426

*E are endothelin receptor antagonists, P5 are phosphodiesterase 5 inhibitors, Pr are prostacyclin analogues. iv ep is intravenous epoprostenol, inh ilp is inhaled iloprost.

§Imputed based on linear model for baseline 6MWD with covariates for Age, Sex, mean STATUS, and mean right arterial pressure.

**Table 4:** Details of included observational studies*

**Reference**	**Jacobs 2009**	**Akagi 2008**	**Channick 2006**	**Hoeper 2003**	**Mathai 2007**	**Hoeper 2004**
**Baseline therapy**	ERA + PDE5i	Pr	ERA	Pr	ERA	None
**Add-on therapy**	Pr	ERA	Pr	ERA	PDE5i	ERA
**Prostacyclin analogue**	intravenous epoprostenol and subcutaneous treprostinil	intravenous epoprostenol	inhaled treprostinil	oral beraprost and inhaled iloprost	NA	NA
**Treatment dose**	10-20 ng/kg/min subcutaneous treprostinil, 38.4 end of observation. 6-8 ng/kg/min intravenous epoprostenol, 16.2 end of observation	62.5 mg bosentan twice daily	6 on 30 mcg inhaled treprostinil 4 daily 6 on 45 mug 4 daily.	62.5 mg bosentan twice daily, increased to 125 mg twice daily after 4 weeks.	20 mg sildenafil (up to 100 mg included) once daily	62.5 mg bosentan twice daily, increased to 125 mg twice daily after 4 weeks.
**Patients at end of study**	10	7	11	20	25	9
**Duration**	16 weeks	1 year	12 weeks	6 months	12 weeks	3 months
**Change 6MWD**	41 (38)	3 (23)	67 (45)	58 (9.6)	20 (28)	57 (35)
**Baseline 6MWD**	387 (30)	392 (16)	339 (26)	346 (23.7)	265 (19)	346
**Age**	37	32	51.2	46	56.2	39
**Sex (% male)**	0.19	0.13	0.09	0.30	0.04	0.22
**STATUS**	3	2	3	3.2	3.1	3.1
**PVR**	957.8¶	776	744	1147	928	1549

*ERA are endothelin receptor antagonists, PDE5i are phosphodiesterase 5 inhibitors, Pr are prostacyclin analogues. iv ep is intravenous epoprostenol, inh ilp is inhaled iloprost, sc trep is subcutaneous treprostinil.

¶Imputed from linear model for PVR based on Age, right arterial pressure, MPAP and cardiac output.

Only two-arm RCTs were identified, with each arm involving the addition of some treatment or placebo to a group of patients who were either treatment naïve or on some baseline treatment. In these studies, included patients had been on the baseline treatment for a time period before randomization (eg. ERA for at least 4 months prior to randomization [42] which was assumed sufficient to derive maximal benefit from the baseline treatment. This assumption implies that any improvement was due to the additional treatment or the placebo effect.

The before-and-after studies were single-arm observational studies which reported the 6MWD of a group of patients on a particular background therapy before and after administering a new treatment. For example, Mathai *et al.* [47] studied the effect of initiating additional PDE5i therapy on a group of patients already on ERA monotherapy, thus providing evidence on the additional benefit of adding PDE5i over ERA alone.

## Methods

**Figure 4:**
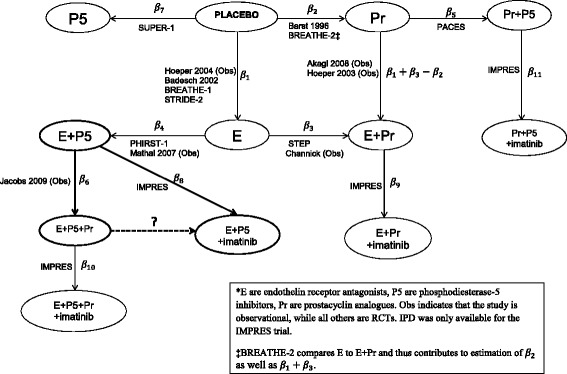
Network of evidence for comparison of effectiveness of monotherapies and combination therapies for PAH. E are endothelin receptor antagonists, P5 are phosphodiesterase-5 inhibitors, Pr are prostacyclin analogues. Obs indicates that the study is observational, while all others are RCTs. IPD was only available for the IMPRES trial.

The final evidence network of observational studies and RCTs for the NMA is shown in Figure 4. The treatment effects are labelled *β*_*i*_ and are the expected short term improvement in 6MWD. Arrow directions indicate the interpretation of these parameters, eg. Positive *β*_2_ means that prostacyclins are more effective than placebo. The network of primary interest is highlighted in bold, and the comparison of primary interest *β*_8_ − *β*_6_, the effectiveness of imatinib against prostacyclins as an add-on to ERA + PDE5i, is highlighted by a bold, dashed, indirect link. This illustrates the necessity of including observational evidence as this network would be disconnected had it been restricted to RCTs. Although this indirect comparison could have been conducted with evidence from only IMPRES and the Jacobs *et al.* studies, the inclusion of a wider range of evidence strengthens our estimates of covariate adjustments and the short term placebo improvements in 6MWD in PAH patients. The following sections explain our development of a NMA model to estimate the parameters *β*_*i*_ by synthesizing all available evidence. As only two-arm RCTs and single-arm observational studies were identified, the models we develop will not be designed for trials with more than two arms. This model development is summarized in Table 5.

**Table 5:** **Summary of NMA models used for comparison of treatment combinations for PAH synthesising aggregate data from the literature and IPD from IMPRES study**

**Model**	**Data**	**Description**	**Section with model details**
M1	Aggregate data only	Aggregates the IPD and includes observational data through random effects on change from baseline 6MWD	2.1
M2	Aggregate and IPD	Extends M1 to combine aggregate data and IPD	2.2
M3	Aggregate and IPD	Extends M2 to include covariate adjustments on change from baseline 6MWD	2.3
M4	Aggregate and IPD	Extends M3 to include interactions between treatment effect and covariates	2.4
S1	Aggregate and IPD	Same as M4 but SE in observational studies are inflated by a factor of 10 to downweight their evidence	3.3
S2	Aggregate and IPD	Same as M4 but constructed a control arm for observational studies where all patients assumed to deteriorate by 25 m from baseline in 6MWD	3.4

### Model M1: network meta-analysis of aggregate data from RCTs and observational studies

The first model we considered was a simple network meta-analysis of aggregated data from the IMPRES study and aggregate data from the literature. The mean short term change in 6MWD for each study *i* and arm *j*, ${\overline{Y}}_{ij}$, was modelled as:

1 $$ {\overline{Y}}_{ij}\sim \mathrm{N}\left({\alpha}_i+{\theta}_{ij},{\mathrm{SE}}_{\mathrm{ij}}\right) $$

where SE_ij_ is the standard error of the observed change in 6MWD in arm *j* of study *i*. It should be noted that this parameterization is slightly different to that used in other network meta-analyses [15,50] as we are using a trial level placebo effect *α*_*i*_ in combination with a trial level effect of treatment, *θ*_*ij*_. The placebo effect is the mean improvement in 6MWD that a group of patients would experience if they entered trial *i* and received only placebo, in addition to their background therapy, and is assumed to be the same for each of the arms of the trial. The effect of treatment in arm *j* is a linear combination of the effects of additional treatments initiated in that arm at the start of the trial:

2 $$ {\theta}_{ij}\sim N\left({f}_{ij}\left(\boldsymbol{\beta} \right),{\sigma}_{\beta}^2\right); $$

*θ*_*ij*_ = effect of additional treatment initiated in *j*^th^ arm of *i*^th^ study

***β*** = vector of treatment effects

*f*_*ij*_ = linear function with coefficients +1 or -1

In Equation (2), random effects with common variance ${\sigma}_{\beta}^2$ were placed on the treatment effects *θ*_*ij*_ in the *i*^th^ study and *j*^th^ arm, as they were assumed to be exchangeable and independent. The entries of the treatment effects vector ***β*** are the treatment effect parameters *β*_*l*_, which we assumed to be fixed effects. For arms receiving only a placebo, it was assumed that *θ*_*iC*_ = 0 so that the improvement in 6MWD is only the placebo effect *α*_*i*_. Two-arm trials with no placebo arm would have mean improvements of *α*_*i*_ + *θ*_*i*1_ and *α*_*i*_ + *θ*_*i*2_, where *θ*_*i*1_ and *θ*_*i*2_ are the effect of the treatment combinations in the first and second arms, respectively.

Observational studies consist of only one arm and their inclusion required an assumption about their *α*_*i*_. We assumed that these *α*_*i*_, the placebo improvement in a trial, which would subsume the placebo effect, would be exchangeable across trials. In the model above, we expressed this by placing a Normal random effect with common mean *α* and variance ${\sigma}_{\alpha}^2$ on the *α*_*i*_ s:

3 $$ {\alpha}_i\sim N\left(\alpha, {\sigma}_{\alpha}^2\right) $$

This use of random effects enables evidence from all RCT and before-and-after observational studies to estimate the expected change in 6MWD. Note that this assumption possibly interferes with randomization as the *α*_*i*_ will be drawn towards the mean *α* and thus the treatment effects ***β*** may be biased. An alternative would be to treat the *α*_*i*_ as fixed effects [24,25,51] and thus preserve randomization, but this would not allow the inclusion of before-and-after studies.

The linear functions *f*_*ij*_() were almost always single values, eg. *β*_6_ for Jacobs *et al.* as the only additional treatment was prostacyclin analogues [43]. In the BREATHE-2 study [40], labelled study *i* for convenience, arm *j* = 1 was a treatment naïve group started on bosentan (ERA) and intravenous epoprostenol (Pr) while arm *j* = 2 was a treatment naïve group started on bosentan (ERA) alone. This was represented by the functions:

$$ {f}_{i1}\left(\boldsymbol{\beta} \right)={\beta}_1+{\beta}_3 $$

$$ {f}_{i2}\left(\boldsymbol{\beta} \right)={\beta}_1 $$

which could be read from Figure 4. The *β*_*i*_ are our analogues of the basic parameters in the standard indirect treatment comparison model described in Dias *et al.* [5], while *f*_*ij*_(***β***) are our analogues of the functional parameters.

The choice of priors for *α* and the *β*_*l*_ s was based on the assumption that no patient would change their walking distance by more than 400 meters, which implied a standard deviation of 200 meters. Assuming that the smallest study had at least 10 patients, this gave $$ SE=\raisebox{1ex}{$200$}\!\left/ \!\raisebox{-1ex}{$\sqrt{10}$}\right. $$ and therefore a prior variance for effects on the mean of *SE*^2^ = 4000. We represented these prior beliefs via Normal distribution, which were judged appropriate in the context of changes in 6MWD through exploratory analysis of the IMPRES data and expert clinical opinion. For $$ {\sigma}_{\beta}^2 $$ and $$ {\sigma}_{\alpha}^2 $$, the vague assumptions that *σ*_*β*_ ≤ 50 meters and *σ*_*α*_ ≤ 50 meters were used, which expressed the belief that individual patients would not differ from the mean improvement in 6MWD by more than 100 meters. Following the recommendation of Lambert *et al.* [52], a uniform prior representing this belief was placed on the standard deviation. These considerations gave the priors:

$$ \alpha \sim N\left(0,4000\right) $$

$$ {\beta}_l\sim N\left(0,4000\right) $$

$$ {\upsigma}_{\upbeta}\sim U\left(0,50\right) $$

$$ {\upsigma}_{\upalpha}\sim U\left(0,50\right) $$

which completed the specification of a NMA model for aggregate data only.

### Model M2: network meta-analysis of IPD and aggregate data from RCTs and observational studies

We extended the aggregate data model described by Equation (1) in Section 2.1 to include individual patient data through the relation:

4 $$ {Y}_{ijk} \sim N\left({\alpha}_i+{\theta}_{ij},{\sigma}^2\right) $$

for the change in 6MWD for patient *k* of arm *j* and study *i*, where *σ*^2^ is a common variance parameter to be fit to the data. Although in general we would use a separate *σ*^2^, with a subscript, for each IPD trial, we have dropped the subscript to simplify the notation as our application only includes a single IPD trial. The treatment effects and placebo effects were as in the aggregate data model M1:

5 $$ {\theta}_{ij} \sim N\left({f}_{ij}\left(\boldsymbol{\beta} \right),{\sigma}_{\beta}^2\right) $$

6 $$ {\alpha}_i\sim N\left(\alpha, {\sigma}_{\alpha}^2\right) $$

Normal prior distributions were again assumed for the means of the Normal distributions and Uniforms were placed on the standard deviations. As in the specification of priors for *α* and the *β*_*l*_ s in model M1, we reasoned that if a patient was assumed not to have an improvement exceeding 400 meters, their standard deviations should be 200 meters and therefore have variances of 40000. These assumptions resulted in the priors:

$$ {\beta}_l\sim N\left(0,40000\right) $$

$$ \sigma \sim U\left(0,50\right). $$

As the evidence for the treatment effects *β*_*l*_ came from both individual patient and aggregate (mean) level data, the ‘vaguer’ prior was used. The prior for the placebo effect *α* and for the standard deviations *σ*_*α*_, and *σ*_*β*_ were kept the same as in the aggregate data models in Section 2.1. This was appropriate as they have the same meaning in both the IPD and aggregate data models.

### Model M3: across-study and within-study covariate adjustments on the placebo effect

To account for across-study heterogeneity, we extended the model to include covariate adjustments on the placebo effects, the *α*_*i*_ s. A further advantage was that these adjustments for differences in the patient populations led to better assessments of the placebo improvement in the single-arm before-and-after studies due to their better explanation of the heterogeneity. We also adjusted for heterogeneity within the studies, which is between-patient heterogeneity, for which we had IPD. The model was defined for a mean covariate ${\overline{X}}_{ij}$ and individual covariate *X*_*ijk*_ as follows:

7 $$ {\overline{Y}}_{ij} \sim N\left({\alpha}_i+\varphi {\overline{X}}_{ij}+{\theta}_{ij},S{E}_{ij}\right) $$

8 $$ {Y}_{ijk} \sim N\left({\alpha}_i+\varphi {\overline{X}}_{ij}+\pi \left({X}_{ijk}-{\overline{X}}_{ij}\right)+{\theta}_{ij},{\sigma}^2\right) $$

9 $$ {\theta}_{ij}\sim N\left({f}_{ij}\left(\boldsymbol{\beta} \right),{\sigma}_{\beta}^2\right) $$

10 $$ {\alpha}_i\sim N\left(\alpha, {\sigma}_{\alpha}^2\right) $$

The last two equations are as in models M1 and M2. In this model, *φ* was the effect of the mean and accounted for across-study differences, while *π* was the effect of an individual’s covariate and accounted for within-study differences.

Note that the difference between *π* and *φ* in Equation (8) quantifies ecological bias, a bias that arises when the effect of the mean of a covariate is different from effect of the covariate itself, and that if *π* = *φ* then there would be no ecological bias.

Priors for *α*, *β*_*l*_, *σ*, *σ*_*α*_, and *σ*_*β*_ were as in the model with no covariate adjustments of Section 2.2, while a vague Normal distribution for mean effects was used for *φ* and a vague Normal distribution for individual effects was used for *π*:

$$ \varphi \sim N\left(0,4000\right) $$

$$ \pi \sim N\left(0,40000\right) $$

which completed the NMA model combining IPD and aggregate data with covariate adjustments on the placebo effect.

### Model M4: within-study covariate adjustments on treatment effects

Our final extension was to include covariate adjustments for the effect of patient characteristics on the efficacy of treatments, the *β*_*l*_ s in the models. Such a model would be useful for predicting efficacy and evaluating cost-effectiveness in patient subgroups with specific baseline characteristics. As only a small number of studies were available in our example for each treatment effect, it was not practical to account for across-study heterogeneity. We therefore restricted treatment effect covariate adjustments to the within-study level, and thus to only the treatment effect of imatinib for which IPD was available. The model was defined as

11 $$ {\overline{Y}}_{ij} \sim N\left({\alpha}_i+\varphi {\overline{X}}_{ij}+{\theta}_{ij},S{E}_{ij}\right) $$

12 $$ {\theta}_{ij} \sim N\left({f}_{ij}\left(\boldsymbol{\beta} \right),{\sigma}_{\beta}^2\right) $$

13 $$ {Y}_{ijk} \sim N\left({\alpha}_i+\varphi {\overline{X}}_{ij}+\pi \left({X}_{ijk}-{\overline{X}}_{ij}\right)+{\theta}_{ijk},{\sigma}^2\right) $$

14 $$ {\theta}_{ijk}\sim N\left({f}_{ij}\left(\boldsymbol{\beta} +\boldsymbol{\gamma} \left({X}_{ijk}-{\overline{X}}_{ij}\right)\right),{\sigma}_{\beta}^2\right) $$

15 $$ {\alpha}_i\sim N\left(\alpha, {\sigma}_{\alpha}^2\right) $$

Where Equations (7) and (14) are modifications of Equations (8) and (2) to include patient specific treatment effects. The elements *γ*_*l*_ of ***γ*** were the effects of the covariate on the treatment effect *β*_*l*_. The linear functions *f*_*ij*_() therefore acted on linear combinations of the treatment effects ***β*** and their covariate adjustments ***γ***.

The same priors as before were used for *α*, *β*_*l*_, *σ*, *σ*_*α*_, *σ*_*β*_, *φ* and *π*, while a Normal distribution for individual patent level effects was used for the *γ*_*l*_, i.e.

$$ {\gamma}_l\sim N\left(0,40000\right) $$

This completed the specification of an NMA model for combining IPD and aggregate data from RCTs and observational studies with covariate adjustments on the placebo and treatment, of imatinib, effect. The models described in these sections are summarized in Table 5 and we applied them to the PAH example.

### Covariate selection via DIC-based forward stepwise selection

Model M4 potentially includes covariates at three different levels and the full model space can be quite large. In our PAH example there are 6 possible covariates, so a total of 2^18^ possible models. Although a model that includes all of these covariates would be highly adjustable to populations in which predictions are desired, it is necessary to avoid over fitting to the data. To avoid over fitting and produce robust predictions, we use the Deviance Information Criterion (DIC, [53]). This is a predictive criterion that balances fit and complexity. It is computationally infeasible to investigate the full model space so we instead apply DIC-based forward stepwise selection [54,55]. This allows us to search through the space of models using the following steps:

1. Initially chosen model has no covariates
2. Fit extended models with one extra covariate from chosen model
3. Choose minimum DIC model from original and extended models.
4. Return to step 2.

Initially, for the PAH example with 6 covariates of interest, Step 2 involves a search of 18 possible models. The second time through involves 17 possible models, and so on. This leads to a maximum of 171 models to search, which is computationally feasible.

## Results

All results presented here are from an implementation of the models described in Section 2, and summarized in Table 5, in the WinBUGS [56] software package. This is a Windows based software for Bayesian inference using Gibbs sampling. The code for these models is provided in Additional file 3 and the authors are happy to respond to any queries about its use. All results were sampled from 250 000 iterations of a single Markov chain Monte Carlo (MCMC) chain following a burn-in of 100 000 iterations. We also sampled a second chain from alternate initial values and confirmed that 250 000 iterations was sufficient for convergence on the basis of the Gelman-Rubin statistic [57].

### Results of models M1 and M2: NMA with no covariate adjustments

**Figure 5:**
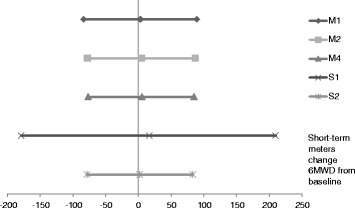
Forest plot of mean and 95% credible interval of posterior distribution for difference in treatment effect of imatinib and Pr given to patients on combination of ERA and PDE5i, on scale of short term change in 6MWD from baseline in meters.

Summary statistics of the posterior distributions of the placebo and treatment effects, on the scale of change in 6MWD in meters, for the comparison of imatinib against prostacyclin analogues as add-on to ERA and PDE5i from the model M1, described in Section 2.1, are presented in Table 6 and Figure 5. This NMA combined only summary statistics from the IMPRES trial and did not make use of the available IPD. The posterior means and 95% credible intervals are comfortably within the prior ranges specified in Section 2.1. The summary of the placebo effect *α* implies that a randomly selected group of patients would be expected to have a mean 6MWD improvement of 4.78 meters, and for this mean to lie within the range of -4.8 and 14.6 meters with a probability of 95%, were they to enter a placebo arm of one of the studies. This is not unreasonable on the basis of the means and standard errors of the observed changes in 6MWD in the control arms of the RCTs, reported in Table 2 and Table 3. The wide and inconclusive 95% credible intervals for the treatment effects and comparison are indicative of the weakness of the evidence. Also provided in Table 6 and Figure 5 are the results of model M2, described in Section 2.2, which combined available aggregate data with IPD from the IMPRES study. The means of the posterior distributions do not change very much but the credible intervals for parameters based on IPD from IMPRES, the *α* (imatinib to E + P5) and treatment effect *β*_8_, shrink. This reduction in the width of the credible intervals is due to the complex interaction between the vague priors in the different parameterisation of model M2 from M1 and is not due to any improvement in the use of the evidence. Even vague priors are somewhat informative and this is illustrated by the reduction in the credible intervals.

**Table 6:** **Results of four network meta-analyses: based on only aggregate data; combining IPD and aggregate data with no covariate adjustments; combining IPD and aggregate data with covariate adjustments for individual patient AGE, baseline STATUS and baseline PVR at within-study level; using the covariate adjusted IPD and aggregate data model with the observational studies down-weighted by inflating their standard errors by a factor of 10; using the covariate adjusted IPD and aggregate data model with constructed control arms for observational studies with an assumed deterioration of 25 meters in 6MWD**

**Model**	**M1**	**M2**	**M3**	**S1**	**S2**
**Model description**	**Aggregate data only**	**IPD and aggregate with no covariate adjustments**	**IPD and aggregate with covariate adjustments**	**Down-weighted observational studies**	**Constructed control arms**
***α***_***i***_ **, Jacobs 2009**	4.77 (-18.60,29.12)	4.55 (-19.07, 29.45)	4.39 (-18.77, 28.59)	4.07 (-17.16, 26.84)	−1.10 (-22.54, 17.77)
***α***_***i***_ **, (imatinib to ERA + PDE5i)**	4.27 (-14.13, 22.59)	3.63 (-10.57, 17.78)	3.50 (-10.59, 17.69)	3.55 (-9.78, 17.12)	0.93 (-11.93, 14.71)
***α*** **(mean of** ***α***_***i***_ **s)**	4.78 (-4.77, 14.60)	4.39 (-5.14, 14.80)	4.30 (-4.73, 14.17)	3.99 (-4.87, 13.68)	0.02 (-8.74, 8.78)
***π***_1_ **(AGE)**	NA	NA	−0.90 (-1.52, -0.27)	−0.90 (-1.52, -0.27)	−0.89 (-1.53, -0.26)
***π***_**3**_ **(STATUS)**	NA	NA	−11.89 (-28.20, 4.44)	−11.92 (-28.29, 4.36)	−12.02 (-28.64, 4.60)
***π***_**5**_ **(PVR)**	NA	NA	−0.05 (-0.70, -0.02)	−0.05 (-0.07, -0.02)	−0.05 (-0.07, -0.02)
***β***_**6**_ **(Pr to ERA + PDE5i)**	35.98 (-44.15, 114.70)	35.16 (-43.81, 113.80)	34.65 (-42.18, 113.20)	22.64 (-168.30, 217.00)	40.65 (-37.09, 117.80)
***β***_**8**_ **(imatinib to ERA + PDE5i)**	39.09 (2.72, 75.80)	39.91 (11.31, 68.36)	40.06 (13.38, 67.67)	39.40 (13.83, 66.03)	42.62 (15.18, 69.79)
**(** ***β***_**8**_ ***− β***_**6**_ **) imatinib v Pr to ERA + PDE5i**	3.11 (-83.70, 89.27)	4.75 (-77.88, 87.10)	5.41 (-76.65, 85.24)	16.76 (-179.26, 209.80)	1.97 (-78.62, 83.53)

Results sampled from 250000 iterations following a burn-in of 100000.

### Results of model M3 and M4: NMA of IPD and aggregate data with covariate adjustments

Summary statistics of the results of the application of the covariate adjusted NMA model of Section 2.3, model M3, to combining IPD from the IMPRES trial with aggregate data from the literature are reported in Table 6 and Figure 5, while further parameter estimates are provided in Additional file 4. We used DIC-based forward stepwise selection to choose the covariate adjustments at across-study and within-study level on the placebo effects and within-study level on the treatment effect of imatinib. It was found that the DIC-minimizing model had no across-study covariate adjustments on the placebo effect but had within-study adjustments for AGE, STATUS and PVR on the placebo effect.

The benefit of including IPD is again indicated by the reduction in the 95% credible interval for the treatment effect of imatinib added to ERA and PDE5i (*β*_8_) from that of model M1 of aggregated data. The 95% credible interval for the indirect comparison of imatinib against prostacyclins as add-on to ERA and PDE5i, (-76.65, 85.24) from model M3, remains approximately the same width as in the aggregate data model, (-83.70, 89.27) from model M1, as illustrated in Figure 5. This is because the effect of additional prostacyclins is based on only aggregate data. The direction of the effects of AGE (-0.90), STATUS (-11.89), and PVR (-0.05) on the expected 6MWD improvement of a patient in the IMPRES trial imply that older and sicker patients have a lower expected improvement, which is reasonable. The non-selection of across-study covariates indicates that the imputed values for missing covariates, such as PVR in Jacobs *et al.*, have no effect on the results. That some values were imputed may affect the DIC-based selection but this is unlikely to be a strong effect as the covariate adjustments were generally found to have little impact.

We further fit model M4, described in Section 2.4, and applied DIC-based stepwise selection to choose covariate adjustments on the treatment effect of imatinib. However, no such covariate adjustments were included so the chosen model M4 was identical to M3.

In addition to applying our NMA methodology to the PAH example, we also tested the impact of its assumptions through sensitivity analyses.

### Sensitivity analysis, model S1: down-weighting the observational studies

In our standard NMA models M1, M2, M3 and M4, we gave equal weight to the results of the before-and-after observational studies and those of two-arm RCTs. An alternative to this assumption is to down-weight the results, recognizing internal bias due to lack of rigor, through a multiplicative adjustment to the standard errors of the results in either or both arms of the aggregate data, i.e.

$$ {\tilde{SE}}_i=\raisebox{1ex}{$S{E}_i$}\!\left/ \!\raisebox{-1ex}{${\delta}_i$}\right. $$

where *δ*_*i*_ is the quality weight of the *i*^th^ study, based on a subjective assessment. This is similar to the weighting of the empirical priors derived from observational evidence discussed in the background section [15,21]. If *δ*_*i*_ = 1, it would represent a study that was judged to be of the highest quality, and its evidence would be given full weight. This was the value we assigned to RCT data. Using the covariate adjusted model of Section 3.2, we repeated the simulations with *δ*_*i*_ = 0.1, increasing observed standard errors by a factor of 10, for the observational studies, thus down-weighting them, relative to RCTs, to represent their poorer quality.

For example, the observed change in 6MWD from baseline in Jacobs *et al.* was 41 meters with a standard error of 38, as reported in Table 4. This sensitivity analysis would assume that this standard error had been 380, substantially larger than any of the observed standard errors reported in Tables 2, 3 or 4 (maximum was about 50). We can therefore conclude that if our analysis is robust to down-weighting by a factor of *δ*_*i*_ = 0.1, it is likely to be robust to most levels of uncertainty we could plausibly observe.

The results from this sensitivity analysis, labeled model S1, are presented in Table 5 and Figure 5. The main change from the results of the model without down-weighting of the observational studies, models M1, M2 and M3 in Table 5, was the increased range of the 95% credible intervals for treatment effects estimated on the basis of observational studies, such as *β*_6_. The range of the 95% credible interval of the comparison of imatinib against prostacyclins as add-on to ERA + PDE5i was also increased, by a large factor, illustrated in Figure 5, due to its reliance on the down-weighted observational studies. The magnitude of the comparative effectiveness (*β*_8_ − *β*_6_) also increased substantially, but is most likely due to the increased random variation illustrated by the expanded credible intervals.

This decrease in the accuracy of the treatment effect estimates and indirect comparisons indicates the influence of the observational studies. As the effect was largely on the accuracy of these estimates and not on their direction, it could be concluded that the NMA methodology was robust to the down-weighting of the observational studies, although its reliance on possibly weak and biased observational evidence was highlighted.

### Sensitivity analysis, model S2: constructed control arms in the observational studies

The lack of control arms in the observational studies presented a difficulty of not knowing what would have happened had patients not been given additional treatment. The NMA models of Section 2 placed Normally distributed random effects on the expected improvements in patients who had entered a study but only received a placebo,

$$ {\alpha}_i\sim N\left(\alpha, {\sigma}_{\alpha}^2\right) $$

An alternative was to construct a control arm for the observational studies by making an assumption about ${\overline{Y}}_{iC}$, the mean change in 6MWD for patients who did not receive additional therapy. As the Jacobs *et al*. study [43] looked at patients who were *deteriorating* on oral therapy, we assumed that patients’ 6MWD would decrease during the trial if they were not given any new treatments. This study included patients whose 6MWD had decreased by 58 meters over a mean time of 20.6 months before entering the study. Our short term follow-up was approximately 24 weeks, which is less than half of 20.6 months, so we assumed a mean change of ${\overline{Y}}_{iC}\approx -25\mathrm{m}$ would be observed in missing control arms over this short term follow-up. We further assumed that the standard error of the mean in this constructed control arm, *SE*_*iC*_, is the same as that observed in the treatment arm. We used these assumptions to construct control arms for all observational studies.

We repeated the analysis using the covariate-adjusted model from Section 3.2 with the constructed control arms, giving the results, labeled model S2, presented in Table 5 and Figure 5. It was difficult to interpret the direction of the change in placebo and treatment effects, due to the effect of covariate adjustments. The direction of the comparison of imatinib against prostacyclins was shifted in favor of prostacyclins, which was expected due to the conservative assumption about the control arms. However, the wide confidence intervals and overall direction of the comparisons remained so the analysis was judged to be robust to this alternative assumption about the control arms.

## Discussion

In this paper we have considered the problem of how to perform a network meta-analysis when the RCT evidence does not form a complete network. Our proposal was to complete the network using single-arm before-and-after observational studies by building a covariate adjusted random effects model on the placebo improvements. We built on recent innovations to construct a model which combines IPD and aggregate data from RCTs and before-and-after studies and allows for the inclusion of covariate adjustments for heterogeneity at across- and within-study level on the placebo effect and within-study level on the treatment effect. Using this model, we performed a clinically novel comparison of the benefit of imatinib against prostacyclins as add-on therapy to PAH patients on a combination of ERA and PDE5i. This comparison was only possible through inclusion of observational studies as an evidence network restricted to RCTs would be disconnected.

As the credible intervals were very wide, the results of our application to PAH were considered to be inconclusive. This was due to the weakness of the evidence as only a few studies, with small sample sizes, were available for each edge of the network. This data limitation may also be the reason why we found that covariate adjustments had little effect on the NMA results and that no across-study adjustments were included on DIC grounds, although we can also interpret this as evidence that heterogeneity had little effect on the NMA. It is possible that important covariates were not reported by IMPRES or other studies, or that reported covariates were incorrectly considered to be of no importance due to the weakness of the data. It is also possible that the stepwise selection algorithm missed important covariates as it only investigates a small portion of the total 2^18^ possible models. A simulation/robustness study could address these concerns but would be computationally intensive as the model selection step, even using stepwise selection to reduce the set of models under consideration, was resource intensive. The best strategy to improve the practical utility of this application of NMA to PAH is to collect further evidence, ideally IPD from a new or existing RCT.

Apart from these data limitations, which are specific to the application, there are a number of limitations and untestable assumptions of the model itself. As in many meta-analysis and NMA models [15], we assumed the effects of particular treatments were the same across studies by placing a fixed effect on each *β*_*l*_. In cases where sufficient data are available, this could be relaxed to a random effects assumption where we assume the *β*_*l*_ from different trials follow a common, possibly Normal, distribution. Our model also assumed, in Equation (2), that effects of additional treatments had the same variance ${\sigma}_{\beta}^2$ in all studies, no matter how many additional treatments were being administered. This is possibly implausible as a the effect of a combination of three new treatments should have a higher variance than the effect of a single new treatment. As in the case of fixed effects, this assumption could be relaxed in cases where sufficient data are available. A further simplification that limits the generalizability of our model is that it is restricted to single- or two-arm trials. To extend the model to trials with three or more arms would require careful consideration of correlation in treatment effects across arms and within studies [5,50].

An assumption of our model that is common to most NMA models is the transitivity or consistency of treatment effects across studies. This is the assumption that studies informing the comparison of treatment A against treatment B and of treatment A against treatment C can be used to inform the comparison of B against C. Our evidence network was sparse and contained only one loop, making it impractical to test for consistency of direct and indirect evidence using node-splitting [58] or other measures of inconsistency [59,60]. If more studies became available, it would be recommended to test that comparing ERA + Pr to ERA using the direct evidence [44,46] gave similar results, within some range of acceptability, to performing the comparison with only indirect evidence.

The principle assumption that allowed the inclusion of single-arm before-and-after observational studies was that the placebo effects, the *α*_*i*_ s, were *exchangeable* or that there was no *a priori* reason that there would be systematic differences between these effects. This assumption allowed us to model the *α*_*i*_ s using a random effects distribution. We recommend the use of this assumption and our model in cases where networks are not densely populated or fully connected when restricted to RCT evidence, such as the PAH example. Decision makers would still need to give a recommendation on which treatment to use in such situations [13] and, indeed, in Australia the PBAC already considers non-randomized observational evidence, particularly in the absence of RCTs [1]. However, this type of evidence is considered to be weak and subject to bias by decision making bodies such as NICE [14]. Additionally, the GRADE scale, which is followed by PBAC, rates the quality of such as evidence as low [61]. In cases where networks can be densely populated and fully connected by RCT evidence, this assumption may shrink placebo effects towards the mean and thus interfere with randomization [24,25,51]. In those cases, we recommend treating the *α*_*i*_ as independent fixed effects, or nuisance parameters, and not including observational evidence.

In the PAH application, clinical opinion supported the assumption of exchangeable placebo effects, although this assumption is not testable statistically. That no across-study adjustments were included in the model selection step gave an indication that there were no systematic differences in these expected improvements and that our exchangeability assumption was warranted. The simple alternative of constructing a control arm for observational studies was investigated but was found to have little effect on the results. We also explored down-weighting the observational evidence and found, as expected, a reduction in the accuracy of our findings, but no change in the overall direction of the indirect comparisons results.

Our model can be criticized on the grounds that the single-arm studies contribute to the estimation of the distribution for the placebo effects *α*_*i*_. We considered an alternative formulation of our model where only the RCTs would contribute to this estimation and the *α*_*i*_ s for the observational studies would be sampled separately from this distribution. This is the method proposed for baseline natural history models by Dias *et al.* [24]. However, our model is designed to be applied to cases where data would already be limited, such as the PAH example, so a further reduction of the evidence base would be undesirable, although in practice the contribution of the observational studies to the *α*_*i*_ estimation will be limited.

A very simple alternative to a random effects assumption for the placebo effects is to use a single fixed effect *α* for the *α*_*i*_ s. This is an assumption that all patient populations started on placebo have the same short term expected improvement in 6MWD and that any differences are due to the treatment or covariate effects. We repeated the NMA with this assumption and found that the results were similar in magnitude and direction to those of the random effects model and that the DIC was considerably higher, with 1889 for fixed effects versus 1870 for random effects. This DIC gives evidence in favor of our random effects model. The single fixed effect model was also not clinically plausible as there were many inherent differences in the studies so a common placebo effect would be difficult to justify.

Several additional sensitivity analyses were conducted. Firstly, as no across-study covariates were included, we applied our final NMA model to an evidence network which included the studies which were excluded due to non-reporting of covariates. This included one extra RCT [62] and three observational studies [63-65]. The results of this sensitivity analysis, not reported in this paper, were almost identical to those of the base case. Prior sensitivity analyses, where we tried prior distributions with greater variances, led us to conclude that the results were not dependent on our choice of prior parameters. Although non-normal priors could be easily implemented if the application required them, normal priors were judged to be appropriate for the continuous outcome of change in 6MWD through expert clinical opinion and exploratory analysis of the IMPRES data.

Aside from the extensions to multi-arm trials, separation of the placebo estimation between RCT and observational studies, and other possibilities so far discussed, there are a variety of directions for future extension of our methodology. One such direction would be to apply the model to non-continuous outcomes such as binary outcomes. NMA models combining IPD and aggregate data for binary outcomes have been discussed in the literature [6,7] and the use of a random effects model for placebo effects to include single-arm studies would be a straightforward extension. Our methods are also readily applicable to pairwise meta-analysis, as it was in this setting that the use of random effects modelling of placebo effects to include single-arm studies was first proposed [23]. Although in pairwise meta-analysis the model would no longer be justified on the grounds of completing evidence networks, it may be useful in cases where there are only a limited number of small RCTs and large, high-quality single-arm studies are available. An additional direction for research is the joint network meta-analysis of multivariate outcomes, such as PVR and change in 6MWD in PAH [66]. This approach would treat all covariates as responses and would account for missing values, a reason for exclusion of several studies, through a form of multiple imputation. This would have the advantage of using the evidence more consistently, rather than our approach of singly imputing missing covariates, such as PVR in Jacobs *et al*. [43]. However, this extension would require a greater evidence base than was available for the PAH example.

All of the limitations we have discussed should be kept in mind if applying our model in order to avoid being misled by the results of an analysis in which observational evidence is included. We would recommend conducting the sensitivity analyses we have described to ensure the model and the implications of its various assumptions are fully understood.

## Conclusions

We have developed an extension of existing NMA methodology to allow the completion of disconnected networks of RCT evidence through the inclusion of single-arm before-and-after observational studies. This model also brings together many recent developments in network meta-analysis of IPD and aggregate data. Our application to PAH demonstrated the utility of our methodology as comparisons impossible to conduct on the basis of RCTs alone could be conducted through the inclusion of observational studies. Although IPD and covariate adjustments were found to make little difference to the results, we believe this model could be easily applied to many other disease areas and settings which require the inclusion of observational evidence. Our work therefore furthers the range of evidence synthesis problems that can be approached through NMA.

## Additional files

Additional file 1:
**PICOS and Search Terms for systematic literature review.**
Additional file 2:
**PRISMA checklist for the systematic literature review.**
Additional file 3:
**WinBUGS code for Model M4: Covariate adjusted NMA of IPD and aggregate data.**
Additional file 4:
**Further parameter estimates from NMA Model M3: Covariate adjusted NMA of IPD and aggregate data.**

## Abbreviations

DIC: Deviance information criterion
ERA: Endothelin receptor antagonist
NMA: Network meta analysis
PAH: Pulmonary arterial hypertension
PDE5i: Phosphodiesterase-5 inhibitors
Pr: Prostacyclin analogues
PVR: Pulmonary vascular resistance
IPD: Individual patient data
RCT: Randomized controlled trial
SE: Standard error
6MWD: 6 minute walk distance
STATUS: World Health Organization New York Health Assessment status
