# Supplementary material for: Network meta-analysis combining individual patient and aggregate data from a mixture of study designs with an application to pulmonary arterial hypertension
Source: BMC Med Res Methodol. 2015 Apr 12;15:34. doi: 10.1186/s12874-015-0007-0 (PMC4403724; doi:10.1186/s12874-015-0007-0)
Supplement: Additional file 1: — PICOS and Search Terms for systematic literature review. [file 12874_2015_7_MOESM1_ESM.docx]

**Additional file 1. PICOS and Search Terms for systematic literature review**

**Population of interest:** Patients with PAH of any severity.

**Interventions:** ERAs, Prostanoids, PDE5is.

**Comparators:** The interventions of interest as combination therapy, or placebo (or any comparator that allows us to create an interlinked network of RCTs to facilitate indirect comparison of the treatments of interest).

**Outcomes:** 6MWD test, change/improvement in World Health Organisation New York Health Assessment functional class, Borg dyspnea score, Haemodynamics, Health Related Quality of Life outcomes, time to clinical worsening, Withdrawals (due to all causes, due to adverse events, due to lack of efficacy).

**Study Design:** Full-text publications of RCTs, case-control studies, and cohort-studies

**Search Terms.**

The search strategy was designed to identify RCTs and observational (cohort and case-control) studies. The search terms were developed using terms used in previous Cochrane reviews and validated study design filters [[67-69](#_ENREF_67)]. The search was limited to studies after 2000 and to those published in English. The search combined ‘disease terms’ with ‘drug names’ and ‘study design terms’ as follows:

**Disease terms**

1. Hypertension, Pulmonary/
2. Pulmonary Heart Disease/
3. (pulmonary adj5 hypertension).ti,ab.
4. 1 or 2 or 3

**Intervention terms**

1. endothelin receptor antagonist/
2. endothelin receptor.ti,ab.
3. (Ambrisentan or Letairis or Volibris).mp. [mp=ti, ab, sh, hw, tn, ot, dm, mf, dv, kw, ps, rs, nm, ui]
4. (Bosentan or Tracleer).mp. [mp=ti, ab, sh, hw, tn, ot, dm, mf, dv, kw, ps, rs, nm, ui]
5. (Prostacyclin or Prostanoid).mp. [mp=ti, ab, sh, hw, tn, ot, dm, mf, dv, kw, ps, rs, nm, ui]
6. (Epoprostenol or Prostacyclin or Flolan or Veletri).mp. [mp=ti, ab, sh, hw, tn, ot, dm, mf, dv, kw, ps, rs, nm, ui]
7. (Treprostinil or Remodulin or Tyvaso).mp. [mp=ti, ab, sh, hw, tn, ot, dm, mf, dv, kw, ps, rs, nm, ui]
8. (Iloprost or Ventavis or Ilomedin or Ilomedine).mp. [mp=ti, ab, sh, hw, tn, ot, dm, mf, dv, kw, ps, rs, nm, ui]
9. Vasodilator agents/
10. phosphodiesterase inhibitors/ or phosphodiesterase 5 inhibitors/
11. ((Phosphodiesterase adj2 inhibitor) or pde5).mp. [mp=ti, ab, sh, hw, tn, ot, dm, mf, dv, kw, ps, rs, nm, ui]
12. (Sildenafil or Revatio).mp. [mp=ti, ab, sh, hw, tn, ot, dm, mf, dv, kw, ps, rs, nm, ui]
13. (Tadalafil or Adcirca).mp. [mp=ti, ab, sh, hw, tn, ot, dm, mf, dv, kw, ps, rs, nm, ui]
14. Or/ 1-13

**Medline randomized controlled trial strategy:**

1. "randomized controlled trial".pt.
2. (random$ or placebo$ or single blind$ or double blind$ or triple blind$).ti,ab.
3. (retraction of publication or retracted publication).pt.
4. or/1-3
5. (animals not humans).sh.
6. ((comment or editorial or meta-analysis or practice-guideline or review or letter or journal correspondence) not "randomized controlled trial").pt.
7. (random sampl$ or random digit$ or random effect$ or random survey or random regression).ti,ab. not "randomized controlled trial".pt.
8. not (5 or 6 or 7)

**Embase randomized controlled trial strategy:**

1. (random$ or placebo$ or single blind$ or double blind$ or triple blind$).ti,ab.
2. RETRACTED ARTICLE/
3. or/1-2
4. (animal$ not human$).sh,hw.
5. (book or conference paper or editorial or letter or review).pt. not exp randomized controlled trial/
6. (random sampl$ or random digit$ or random effect$ or random survey or random regression).ti,ab. not exp randomized controlled trial/
7. not (4 or 5 or 6)

**Medline cohort and case-control strategy:**

1. exp cohort studies/
2. cohort$.tw.
3. controlled clinical trial.pt.
4. epidemiologic methods/
5. limit 4 to yr=1966-1989
6. exp case-control studies/
7. (case$ and control$).tw.
8. or/1-3,5-7

**Embase cohort and case-control strategy:**

1. exp cohort analysis/
2. exp longitudinal study/
3. exp prospective study/
4. exp follow up/
5. cohort$.tw.
6. exp case control study/
7. (case$ and control$).tw.
8. or/1-7

Legend:

/ after an index term indicates that all subheadings were selected.

* before an index term indicates that that term was focused - i.e. limited to records where major MeSH/Emtree term.

"exp" before an index term indicates that the term was exploded.

.tw. indicates a search for a term in title/abstract.

.mp. indicates a free text search for a term.

.pt. indicates a search for a publication type.

$ at the end of a term indicates that this term has been truncated.

? in the middle of a term indicates the use of a wildcard.

adj indicates a search for two terms where they appear adjacent to one another.

sh indicates a search term for subheading.
