# Supplementary material for: Network meta-analysis combining individual patient and aggregate data from a mixture of study designs with an application to pulmonary arterial hypertension
Source: BMC Med Res Methodol. 2015 Apr 12;15:34. doi: 10.1186/s12874-015-0007-0 (PMC4403724; doi:10.1186/s12874-015-0007-0)
Supplement: Additional file 2: — PRISMA checklist for the systematic literature review. [file 12874_2015_7_MOESM2_ESM.docx]

**Additional file 2. PRISMA checklist for the systematic literature review.**

As noted in Section 1.1, the application of the methodology to PAH is for illustrative purposes only and should not be used to guide clinical practice as the model relies on a number of assumptions necessary to facilitate the indirect comparison. Certain items, such as search protocols and risk of bias assessments, have not been presented in the interest of brevity and to keep a methodological focus.

| Section/topic | Item No | Checklist item | Reported on page No |
| --- | --- | --- | --- |
| **Title** | | | |
| Title | 1 | Identify the report as a systematic review, meta-analysis, or both | Page 2 (Methods) |
| **Abstract** | | | |
| Structured summary | 2 | Provide a structured summary including, as applicable, background, objectives, data sources, study eligibility criteria, participants, interventions, study appraisal and synthesis methods, results, limitations, conclusions and implications of key findings, systematic review registration number | Page 2 (Abstract) |
| **Introduction** | | | |
| Rationale | 3 | Describe the rationale for the review in the context of what is already known | Page 5 (Section 1.1) |
| Objectives | 4 | Provide an explicit statement of questions being addressed with reference to participants, interventions, comparisons, outcomes, and study design (PICOS) | Appendix 1 |
| **Methods** | | | |
| Protocol and registration | 5 | Indicate if a review protocol exists, if and where it can be accessed (such as web address), and, if available, provide registration information including registration number | Does not exist (application is illustrative only) |
| Eligibility criteria | 6 | Specify study characteristics (such as PICOS, length of follow-up) and report characteristics (such as years considered, language, publication status) used as criteria for eligibility, giving rationale | Appendix 1 |
| Information sources | 7 | Describe all information sources (such as databases with dates of coverage, contact with study authors to identify additional studies) in the search and date last searched | Page 6 (Systematic literature review of studies in literature) |
| Search | 8 | Present full electronic search strategy for at least one database, including any limits used, such that it could be repeated | Appendix 1 |
| Study selection | 9 | State the process for selecting studies (that is, screening, eligibility, included in systematic review, and, if applicable, included in the meta-analysis) | Appendix 1 |
| Data collection process | 10 | Describe method of data extraction from reports (such as piloted forms, independently, in duplicate) and any processes for obtaining and confirming data from investigators | Not presented (application is illustrative only) |
| Data items | 11 | List and define all variables for which data were sought (such as PICOS, funding sources) and any assumptions and simplifications made | Appendix 1 and page 6. |
| Risk of bias in individual studies | 12 | Describe methods used for assessing risk of bias of individual studies (including specification of whether this was done at the study or outcome level), and how this information is to be used in any data synthesis | Page 6 |
| Summary measures | 13 | State the principal summary measures (such as risk ratio, difference in means). | Page 5  (Section 1.1) |
| Synthesis of results | 14 | Describe the methods of handling data and combining results of studies, if done, including measures of consistency (such as I^2^ statistic) for each meta-analysis | Pages 7-11 (Section 2) |
| Risk of bias across studies | 15 | Specify any assessment of risk of bias that may affect the cumulative evidence (such as publication bias, selective reporting within studies) | Page 13 (Section 3.3) |
| Additional analyses | 16 | Describe methods of additional analyses (such as sensitivity or subgroup analyses, meta-regression), if done, indicating which were pre-specified | Page 13-14 (Sections 3.3 and 3.4) |
| **Results** | | | |
| Study selection | 17 | Give numbers of studies screened, assessed for eligibility, and included in the review, with reasons for exclusions at each stage, ideally with a flow diagram | Flow diagrams in Figures 2 and 3. |
| Study characteristics | 18 | For each study, present characteristics for which data were extracted (such as study size, PICOS, follow-up period) and provide the citations | Tables 2, 3 and 4 |
| Risk of bias within studies | 19 | Present data on risk of bias of each study and, if available, any outcome-level assessment (see item 12). | RCT checklist not presented (application is illustrative only) |
| Results of individual studies | 20 | For all outcomes considered (benefits or harms), present for each study (a) simple summary data for each intervention group and (b) effect estimates and confidence intervals, ideally with a forest plot | Tables 2, 3 and 4. |
| Synthesis of results | 21 | Present results of each meta-analysis done, including confidence intervals and measures of consistency | Table 6 and Appendix 2 |
| Risk of bias across studies | 22 | Present results of any assessment of risk of bias across studies (see item 15) | Not presented (application is illustrative only) |
| Additional analysis | 23 | Give results of additional analyses, if done (such as sensitivity or subgroup analyses, meta-regression) (see item 16) | Table 6. |
| **Discussion** | | | |
| Summary of evidence | 24 | Summarise the main findings including the strength of evidence for each main outcome; consider their relevance to key groups (such as health care providers, users, and policy makers) | Pages 14-16 (Discussion) |
| Limitations | 25 | Discuss limitations at study and outcome level (such as risk of bias), and at review level (such as incomplete retrieval of identified research, reporting bias) | Pages 15-16 (Discussion) |
| Conclusions | 26 | Provide a general interpretation of the results in the context of other evidence, and implications for future research | Page 15 (Discussion) and Page 17 (Conclusions) |
| **Funding** | | | |
| Funding | 27 | Describe sources of funding for the systematic review and other support (such as supply of data) and role of funders for the systematic review | Page 18 (Acknowledgements) |
