# Supplementary material for: Network meta-analysis combining individual patient and aggregate data from a mixture of study designs with an application to pulmonary arterial hypertension
Source: BMC Med Res Methodol. 2015 Apr 12;15:34. doi: 10.1186/s12874-015-0007-0 (PMC4403724; doi:10.1186/s12874-015-0007-0)
Supplement: Additional file 3: — WinBUGS code for Model M4: Covariate adjusted NMA of IPD and aggregate data. [file 12874_2015_7_MOESM3_ESM.docx]

**Additional file 3. WinBUGS code for Model M4: Covariate adjusted NMA of IPD and aggregate data**

Our WinBUGS [[56](#_ENREF_56)] code for the model M3 described in Section 2.3, with comments and selected inputs, is provided below. Omitting the covariates and or only using AD will obtain models M1 and M2. Covariates are omitted or included by setting *across.cov[j]*, *within.cov[j]*, or *inter.cov[j]* to 0 or 1, respectively, where *j* numbers the possible covariates, six in this example. Appropriately setting *Naggregate* or *Nipd*, the number of aggregate or ipd studies, respectively, will set the analysis to AD, IPD or a combination of both.

As the IPD was proprietary, only the AD of the studies provided below is real while the IPD is simulated. For the IPD, we generated four two-arm trials with five patients in each arm and investigating the treatment combinations of the original IMPRES study. The covariate values for age, sex, baseline walk distance, baseline MPAP, baseline NYHA STATUS and baseline PVR were generated from distributions similar to those of the original IPD. However, re-running the analyses will not generate similar results to those presented in the Table 6 as our simulated data assumed no relation between treatments and outcomes and is merely illustrative. Several tricks were employed to implement the model and are explained below. We are happy to answer any queries about our code.

The WinBUGS language does not have the ‘if’ and ‘else’ construction commonly found in other programming languages. We overcame this absence through the use of intermediary and indicator variables. For example, if study *i* was observational, indicated by *observational[i]=1*, we did not want to include its control arm data *YC[i]* (the short term change in 6MWD). We would like to able to write (in WinBUGS pseudo-code)

*if(!observational[j])YC[j]~dnorm(muC[j],tauC[j]*

so that the model parameter *muC[j*] was only connected to the data *YC[j]* if the study was not observational. We accomplish the same functionality through an intermediary variable *muCtemp[j]* which is always connected to the data but is only equal to *muC[j]* if the study is not observational, i.e.

*YC[j]~dnorm(muCtemp[j],tauC[j])*

*muCtemp[j]<-(1-observational[j])*muC[j]*

This method is also used for conditional inclusion of covariate effects. For example, within-study covariate effects *pi[j]*, for covariate *j*, were included only if the indicator *within.cov[j]* was *1* and this was linked to *pi[j]* through the intermediary variable *pi.temp[j]*.

One further note is that WinBUGS cannot manage very long program lines, so we broke our equations across several lines, for example in the covariate effect equations.

*# Network Meta regression model including IPD and aggregate data studies*

*# Includes covariate effects and interaction terms*

*model*

*{*

*# Aggregate data from literature review*

*for (j in 1:Naggregate) { # Naggregate is number of included aggregate data studies*

*# delta are the quality weights (delta=1 means no internal bias, delta=0.1 is our down-weighting factor for model S1)*

*tauA[j] <- deltaA[j]/(seA[j] * seA[j]) # precision in alternate arm*

*tauC[j] <- deltaC[j]/(seC[j] * seC[j]) # precision in control arm*

*YA[j] ~ dnorm(muA[j], tauA[j]) # Change from baseline in alternate arm*

*YC[j] ~ dnorm(muCtemp[j], tauC[j]) # Change from baseline in control arm*

*muCtemp[j] <- (1 - observational[j]) * muC[j] # If the study is observational, muC[j] is not connected to the (missing) data*

*muA[j] <- alpha[j] + phi[1] * mean.age[2, j] + phi[2] * mean.sex[2, j] + phi[3] * mean.status[2, j] + phi[4] * mean.walk[2, j] + phi[5] * mean.pvr[2, j] + theta[1, j] + phi[6] * mean.mpap[2, j]*

*muC[j] <- alpha[j] + phi[1] * mean.age[1, j] + phi[2] * mean.sex[1, j] + phi[3] * mean.status[1, j] + phi[4] * mean.walk[1, j] + phi[5] * mean.pvr[1, j] + theta.control[1, j] + phi[6] * mean.mpap[1, j]*

*}*

*# Prior is on the standard deviation scale*

*tau <- 1/(sd * sd)*

*sd ~ dunif(0.00000E+00, upper.lsd)*

*# IPD data in paper is from IMPRES study but here is only simulated*

*for (j in 1:Nipd) {# Nipd is number of included IPD studies*

*for (i in 1:N[j]) {# N[j] is number of patients in study j*

*Y[i, j] ~ dnorm(mu[i, j], tau) # Change from baseline 6MWD for patient i*

*# It was necessary to split the (long) model equation for WinBUGS to work*

*mu[i, j] <- alpha[j + Naggregate] + theta[i, j + Naggregate] * (treat[i, j] - 1) + theta.control[i, j + Naggregate] * treat[i, j] + covariates.across[i, j] + covariates.within[i, j]*

*# Across-study covariate effects on the main effect*

*covariates.across[i, j] <- phi[1] * mean.age[treat[i, j], j + Naggregate] + phi[2] * mean.sex[treat[i, j], j + Naggregate] + phi[3] * mean.status[treat[i, j], j + Naggregate] + phi[4] * mean.walk[treat[i, j], j + Naggregate] + phi[5] * mean.pvr[treat[i, j], j + Naggregate] + phi[6] * mean.mpap[treat[i, j], j + Naggregate]*

*# Within-study covariate effects on the main effect*

*covariates.within[i, j] <- pi[1] * (age[i, j] - mean.age[treat[i, j], j + Naggregate]) + pi[2] * (sex[i, j] - mean.sex[treat[i, j], j + Naggregate]) + pi[3] * (status[i, j] -mean.status[treat[i, j], j + Naggregate]) + pi[4] * (walk[i, j] - mean.walk[treat[i, j], j + Naggregate]) + pi[5] * (pvr[i, j] - mean.pvr[treat[i, j], j + Naggregate]) + pi[6] * (mpap[i, j] - mean.mpap[treat[i, j], j + Naggregate])*

*}*

*}*

*# Effect of additional treatments in aggregate studies*

*# beta[i] are the treatment effects*

*# study.beta[i,j] indicates whether treatment i was used as add-on therapy in study j*

*for (j in 1:Naggregate) {*

*theta.mean.agg[1, j] <- beta[1] * study.beta[1, j]*

*theta.control.mean.agg[1, j] <- beta[1] * study.beta.control[1, j]*

*for (i in 2:N.beta) {*

*theta.mean.agg[i, j] <- theta.mean.agg[i - 1, j] + beta[i] * study.beta[i, j]*

*theta.control.mean.agg[i, j] <- theta.control.mean.agg[i - 1, j] + beta[i] * study.beta.control[i, j]*

*}*

*theta[1, j] ~ dnorm(theta.mean.agg[N.beta, j], beta.tau)*

*theta.control[1, j] ~ dnorm(theta.control.mean.agg[N.beta, j], beta.tau)*

*}*

*for (j in (Naggregate + 1):(Nipd + Naggregate)) {*

*for (i in 1:N[j - Naggregate]) {*

*theta[i, j] ~ dnorm(theta.mean.ipd[N.beta, i, j], beta.tau)*

*theta.control[i, j] ~ dnorm(theta.control.mean.ipd[N.beta, i, j], beta.tau)*

*# Place all within-study treatment covariate interactions here*

*theta.mean.ipd[1, i, j] <- study.beta[1, j] * (beta[1] + gamma[1, 1] * (age[i, j - Naggregate] - mean.age[treat[i, j - Naggregate], j]) + gamma[1, 2] * (sex[i, j - Naggregate] - mean.sex[treat[i, j - Naggregate], j]) + gamma[1, 3] * (status[i, j - Naggregate] - mean.status[treat[i, j - Naggregate], j]) + gamma[1, 4] * (walk[i, j - Naggregate] - mean.walk[treat[i, j - Naggregate], j]) + gamma[1, 5] * (pvr[i, j - Naggregate] - mean.pvr[treat[i, j - Naggregate], j]) + gamma[1, 6] * (mpap[i, j - Naggregate] - mean.mpap[treat[i, j - Naggregate], j]))*

*theta.control.mean.ipd[1, i, j] <- study.beta.control[1, j] * (beta[1] + gamma[1, 1] * (age[i, j - Naggregate] - mean.age[treat[i, j - Naggregate], j]) + gamma[1, 2] * (sex[i, j - Naggregate] - mean.sex[treat[i, j - Naggregate], j]) + gamma[1, 3] * (status[i, j - Naggregate] - mean.status[treat[i, j - Naggregate], j]) + gamma[1, 4] * (walk[i, j - Naggregate] - mean.walk[treat[i, j - Naggregate], j]) + gamma[1, 5] * (pvr[i, j - Naggregate] - mean.pvr[treat[i, j - Naggregate], j]) + gamma[1, 6] * (mpap[i, j - Naggregate] - mean.mpap[treat[i, j - Naggregate], j]))*

*for (k in 2:N.beta) {*

*theta.mean.ipd[k, i, j] <- theta.mean.ipd[k - 1, i, j] + study.beta[k, j] * (beta[k] + gamma[k, 1] * (age[i, j - Naggregate] - mean.age[treat[i, j - Naggregate], j]) + gamma[k, 2] * (sex[i, j - Naggregate] - mean.sex[treat[i, j - Naggregate], j]) + gamma[k, 3] * (status[i, j - Naggregate] - mean.status[treat[i, j - Naggregate], j]) + gamma[k, 4] * (walk[i, j - Naggregate] - mean.walk[treat[i, j - Naggregate], j]) + gamma[k, 5] * (pvr[i, j - Naggregate] - mean.pvr[treat[i, j - Naggregate], j]) + gamma[k, 6] * (mpap[i, j - Naggregate] - mean.mpap[treat[i, j - Naggregate], j]))*

*theta.control.mean.ipd[k, i, j] <- theta.control.mean.ipd[k - 1, i, j] + study.beta.control[k, j] * (beta[k] + gamma[k, 1] * (age[i, j - Naggregate] - mean.age[treat[i, j - Naggregate], j]) + gamma[k, 2] * (sex[i, j - Naggregate] - mean.sex[treat[i, j - Naggregate], j]) + gamma[k, 3] * (status[i, j - Naggregate] - mean.status[treat[i, j - Naggregate], j]) + gamma[k, 4] * (walk[i, j - Naggregate] - mean.walk[treat[i, j - Naggregate], j]) + gamma[k, 5] * (pvr[i, j - Naggregate] - mean.pvr[treat[i, j - Naggregate], j]) + gamma[k, 6] * (mpap[i, j - Naggregate] - mean.mpap[treat[i, j - Naggregate], j]))*

*}*

*}*

*}*

*# Random effects on the baseline change in 6MWD (random.effects=1 if using a random effect, otherwise uses fixed effect alpha.mean)*

*for (j in 1:(Nipd + Naggregate)) {*

*alpha.random.effect[j] ~ dnorm(alpha.mean, alpha.tau)*

*alpha[j] <- (1 - random.effects) * alpha.mean + random.effects **

*alpha.random.effect[j]*

*}*

*# Prior on baseline change in 6MWD*

*alpha.mean ~ dnorm(0.00000E+00, mean.vague.prec)*

*# Place a uniform prior on the sd of alpha, as advised by Lambert et al*

*alpha.tau <- 1/(alpha.sd * alpha.sd)*

*alpha.sd ~ dunif(0.00000E+00, upper.lsd)*

*# Priors for the treatment effects (with random effects)*

*for (j in 1:N.beta) {*

*beta[j] ~ dnorm(0.00000E+00, vague.prec)*

*}*

*# Use a uniform prior on the standard deviation*

*beta.tau <- 1/(beta.sd * beta.sd)*

*beta.sd ~ dunif(0.00000E+00, upper.lsd)*

*# Priors for all 6 possible covariate effects*

*for (j in 1:6) {*

*pi.temp[j] ~ dnorm(0.00000E+00, vague.prec) # Within study effect*

*pi[j] <- within.cov[j] * pi.temp[j] #pi[j] is 0 if effect is not included at within-study level*

*phi.temp[j] ~ dnorm(0.00000E+00, mean.vague.prec) # Effect of study mean*

*phi[j] <- across.cov[j] * phi.temp[j] # phi[j] is 0 if effect is not included at across-study level*

*}*

*# Priors for the 6 possible treatment covariate interaction effects at within-study level*

*for (j in 1:N.beta) {*

*for (i in 1:6) {*

*gamma.temp[j, i] ~ dnorm(0.00000E+00, vague.prec)*

*gamma[j, i] <- inter.cov[i] * gamma.temp[j, i] # gamma[j,i] is 0 if effect not included*

*}*

*}*

*}*

*##############################################################################################################*

*## Two sets of initial values for placebo effects ###############################*

*list(alpha=c(0.00000E+00, 0.00000E+00, 0.00000E+00, 0.00000E+00, 0.00000E+00, 0.00000E+00, 0.00000E+00, 0.00000E+00, 0.00000E+00, 0.00000E+00, 0.00000E+00, 0.00000E+00, 0.00000E+00, 0.00000E+00, 0.00000E+00, 0.00000E+00, 0.00000E+00, 0.00000E+00, 0.00000E+00))*

*list(alpha=c(0.00000E+00, 0.00000E+00, 0.00000E+00, 0.00000E+00, 0.00000E+00, 0.00000E+00, 0.00000E+00, 0.00000E+00, 0.00000E+00, 0.00000E+00, 0.00000E+00, 0.00000E+00, 0.00000E+00, 0.00000E+00, 0.00000E+00, 0.00000E+00, 0.00000E+00, 0.00000E+00, 0.00000E+00))*

*##################################################################################################################*

*## Illustrative data – AD is real while IPD is simulated* ##############################

*list(Naggregate=1.50000E+01, Nipd=4.00000E+00, Y= structure(.Data= c(-9.00000E+01, 1.00000E+00, 2.30000E+01, 5.90000E+01, 5.00000E+01, 6.50000E+01, 1.04000E+02, 1.04000E+02, 6.00000E+01, 1.04000E+02, -6.00000E+00, -5.00000E+01, -3.40000E+01, 1.00000E+01, 3.00000E+01, -2.40000E+01, 6.00000E+00, 4.70000E+01, 8.40000E+01, 3.20000E+01, -1.20000E+01, 1.20000E+01, -4.50000E+01, -5.40000E+01, -2.40000E+01, -2.20000E+01, 9.10000E+01, 6.60000E+01, 4.20000E+01, -8.70000E+01, 6.00000E+00, 9.10000E+01, 1.00000E+01, -1.40000E+01, 3.80000E+01, -5.20000E+01, -5.80000E+01, 8.00000E+01, 6.60000E+01, -6.60000E+01), .Dim=c(10, 4)), treat= structure(.Data= c(1.00000E+00, 1.00000E+00, 1.00000E+00, 1.00000E+00, 1.00000E+00, 1.00000E+00, 1.00000E+00, 1.00000E+00, 1.00000E+00, 1.00000E+00, 1.00000E+00, 1.00000E+00, 1.00000E+00, 1.00000E+00, 1.00000E+00, 1.00000E+00, 1.00000E+00, 1.00000E+00, 1.00000E+00, 1.00000E+00, 2.00000E+00, 2.00000E+00, 2.00000E+00, 2.00000E+00, 2.00000E+00, 2.00000E+00, 2.00000E+00, 2.00000E+00, 2.00000E+00, 2.00000E+00, 2.00000E+00, 2.00000E+00, 2.00000E+00, 2.00000E+00, 2.00000E+00, 2.00000E+00, 2.00000E+00, 2.00000E+00, 2.00000E+00, 2.00000E+00), .Dim=c(10, 4)), N=c(1.00000E+01, 1.00000E+01, 1.00000E+01, 1.00000E+01), age= structure(.Data= c(6.20000E+01, 7.20000E+01, 3.30000E+01, 5.30000E+01, 5.80000E+01, 3.40000E+01, 8.20000E+01, 5.20000E+01, 4.20000E+01, 4.90000E+01, 3.90000E+01, 3.90000E+01, 5.10000E+01, 5.60000E+01, 7.50000E+01, 3.00000E+01, 4.10000E+01, 4.40000E+01, 4.90000E+01, 3.50000E+01, 7.20000E+01, 2.90000E+01, 4.20000E+01, 4.50000E+01, 2.10000E+01, 3.80000E+01, 4.80000E+01, 3.50000E+01, 8.50000E+01, 6.00000E+01, 5.80000E+01, 5.80000E+01, 6.40000E+01, 4.00000E+01, 4.80000E+01, 2.00000E+01, 5.50000E+01, 2.80000E+01, 6.60000E+01, 5.10000E+01), .Dim=c(10, 4)), sex= structure(.Data= c(1.00000E+00, 0.00000E+00, 0.00000E+00, 0.00000E+00, 0.00000E+00, 0.00000E+00, 0.00000E+00, 1.00000E+00, 0.00000E+00, 0.00000E+00, 1.00000E+00, 0.00000E+00, 0.00000E+00, 1.00000E+00, 0.00000E+00, 0.00000E+00, 0.00000E+00, 0.00000E+00, 1.00000E+00, 0.00000E+00, 0.00000E+00, 0.00000E+00, 1.00000E+00, 0.00000E+00, 0.00000E+00, 0.00000E+00, 0.00000E+00, 0.00000E+00, 0.00000E+00, 0.00000E+00, 0.00000E+00, 0.00000E+00, 0.00000E+00, 0.00000E+00, 0.00000E+00, 0.00000E+00, 0.00000E+00, 0.00000E+00, 0.00000E+00, 0.00000E+00), .Dim=c(10, 4)), mpap= structure(.Data= c(4.70000E+01, 6.00000E+01, 4.80000E+01, 5.60000E+01, 8.70000E+01, 7.10000E+01, 6.50000E+01, 3.10000E+01, 5.80000E+01, 4.80000E+01, 6.50000E+01, 7.30000E+01, 5.70000E+01, 4.90000E+01, 5.30000E+01, 4.50000E+01, 7.30000E+01, 5.80000E+01, 7.20000E+01, 4.10000E+01, 7.40000E+01, 3.70000E+01, 4.70000E+01, 6.00000E+01, 4.70000E+01, 7.10000E+01, 6.10000E+01, 4.90000E+01, 5.10000E+01, 5.20000E+01, 5.10000E+01, 6.30000E+01, 6.60000E+01, 3.90000E+01, 5.50000E+01, 2.80000E+01, 9.20000E+01, 5.80000E+01, 6.20000E+01, 5.30000E+01), .Dim=c(10, 4)), status= structure(.Data= c(2.00000E+00, 2.00000E+00, 2.00000E+00, 3.00000E+00, 2.00000E+00, 3.00000E+00, 3.00000E+00, 2.00000E+00, 3.00000E+00, 4.00000E+00, 3.00000E+00, 3.00000E+00, 3.00000E+00, 4.00000E+00, 3.00000E+00, 2.00000E+00, 3.00000E+00, 3.00000E+00, 3.00000E+00, 3.00000E+00, 2.00000E+00, 3.00000E+00, 3.00000E+00, 3.00000E+00, 3.00000E+00, 3.00000E+00, 3.00000E+00, 3.00000E+00, 2.00000E+00, 3.00000E+00, 4.00000E+00, 3.00000E+00, 2.00000E+00, 2.00000E+00, 3.00000E+00, 3.00000E+00, 4.00000E+00, 3.00000E+00, 3.00000E+00, 4.00000E+00), .Dim=c(10, 4)), walk= structure(.Data= c(4.14000E+02, 2.90000E+02, 3.95000E+02, 3.78000E+02, 3.29000E+02, 4.23000E+02, 2.46000E+02, 3.30000E+02, 2.36000E+02, 2.47000E+02, 5.56000E+02, 3.45000E+02, 4.51000E+02, 4.47000E+02, 3.79000E+02, 4.25000E+02, 2.68000E+02, 2.76000E+02, 3.84000E+02, 2.26000E+02, 4.83000E+02, 3.03000E+02, 2.89000E+02, 3.43000E+02, 4.20000E+02, 4.66000E+02, 3.30000E+02, 3.33000E+02, 3.28000E+02, 3.83000E+02, 3.18000E+02, 3.80000E+02, 2.27000E+02, 3.49000E+02, 3.18000E+02, 3.63000E+02, 5.04000E+02, 2.55000E+02, 3.33000E+02, 4.54000E+02), .Dim=c(10, 4)), pvr= structure(.Data= c(1.19700E+03, 9.51000E+02, 1.63000E+03, 5.55000E+02, 1.56500E+03, 1.01600E+03, 4.15000E+02, 1.41000E+03, 1.10200E+03, 1.59800E+03, 8.79000E+02, 1.26600E+03, 1.23800E+03, 1.47900E+03, 1.31000E+03, 5.10000E+02, 1.08600E+03, 2.98000E+02, 1.05000E+03, 6.44000E+02, 1.23700E+03, 1.36400E+03, 1.34900E+03, 1.26300E+03, 1.75700E+03, 1.23500E+03, 9.40000E+01, 1.28800E+03, 4.52000E+02, 6.94000E+02, 6.45000E+02, 7.52000E+02, 1.12500E+03, 1.83400E+03, 5.19000E+02, 1.14700E+03, 1.16300E+03, 1.19100E+03, 1.23800E+03, 1.61500E+03), .Dim=c(10, 4)), YA=c(4.10000E+01, 3.00000E+00, 6.70000E+01, 5.80000E+01, 1.96000E+01, 5.70000E+01, 4.02000E+01, 2.98000E+01, 3.00000E+01, 7.20000E+01, 7.00000E+01, 3.60000E+01, 2.30000E+01, 3.20000E+01, 5.00000E+01, -8.40000E+00, -6.20000E+00, 3.12000E+01, -3.00000E+00), YC=c(-2.50000E+01, -2.50000E+01, -2.50000E+01, -2.50000E+01, -2.50000E+01, -2.50000E+01, 1.88000E+01, 1.00000E+00, 4.00000E+00, 4.60000E+01, -6.00000E+00, -8.00000E+00, -6.50000E+00, -1.50000E+01, 2.00000E+00, -1.60000E+00, 4.54000E+01, 4.70000E+01, 2.42000E+01), seA=c(3.78021E+01, 2.29824E+01, 4.47590E+01, 9.61509E+00, 2.84582E+01, 3.45704E+01, 8.50000E+00, 5.31322E+00, 1.02899E+01, 1.14708E+01, 2.34135E+01, 6.54535E+00, 9.32059E+00, 2.47588E+01, 9.00000E+00, 1.67260E+01, 2.70193E+01, 2.37432E+01, 3.35917E+01), seC=c(3.78021E+01, 2.29824E+01, 4.47590E+01, 9.61509E+00, 2.84582E+01, 3.45704E+01, 9.15000E+00, 5.29336E+00, 1.06187E+01, 1.96061E+01, 5.04705E+01, 9.45560E+00, 9.16903E+00, 3.32415E+01, 5.00000E+00, 2.77301E+01, 1.87686E+01, 2.03617E+01, 2.78108E+01), mean.age= structure(.Data= c(3.70000E+01, 3.20000E+01, 5.12000E+01, 4.60000E+01, 5.61600E+01, 3.90000E+01, 5.17000E+01, 4.75000E+01, 5.10000E+01, 4.70000E+01, 4.74000E+01, 4.72000E+01, 5.30000E+01, 4.00000E+01, 4.90000E+01, 5.08000E+01, 5.10000E+01, 5.56000E+01, 4.18000E+01, 3.70000E+01, 3.20000E+01, 5.12000E+01, 4.60000E+01, 5.61600E+01, 3.90000E+01, 5.00000E+01, 4.78000E+01, 4.90000E+01, 4.50000E+01, 5.22000E+01, 4.87000E+01, 4.90000E+01, 4.00000E+01, 4.80000E+01, 5.94000E+01, 3.90000E+01, 5.24000E+01, 4.18000E+01), .Dim=c(2, 19)), mean.sex= structure(.Data= c(1.87500E-01, 1.25000E-01, 9.00000E-02, 3.00000E-01, 4.00000E-02, 2.22222E-01, 2.20000E-01, 2.30000E-01, 2.10000E-01, 4.50000E-01, 0.00000E+00, 2.20000E-01, 2.40000E-01, 3.00000E-01, 1.90000E-01, 2.00000E-01, 2.00000E-01, 4.00000E-01, 2.00000E-01, 1.87500E-01, 1.25000E-01, 9.00000E-02, 3.00000E-01, 4.00000E-02, 2.22222E-01, 2.10000E-01, 1.80000E-01, 2.10000E-01, 2.30000E-01, 1.90000E-01, 2.10000E-01, 2.20000E-01, 2.40000E-01, 2.10000E-01, 0.00000E+00, 0.00000E+00, 2.00000E-01, 0.00000E+00), .Dim=c(2, 19)), mean.mpap= structure(.Data= c(5.60000E+01, 8.01000E+01, 4.90000E+01, 5.50000E+01, 5.50800E+01, 6.20000E+01, 5.14534E+01, 5.11000E+01, 5.20000E+01, 6.09000E+01, 5.60000E+01, 5.30000E+01, 4.90000E+01, 5.90000E+01, 5.60000E+01, 6.44000E+01, 5.72000E+01, 6.06000E+01, 4.92000E+01, 5.60000E+01, 8.01000E+01, 4.90000E+01, 5.50000E+01, 5.50800E+01, 6.20000E+01, 5.14534E+01, 5.22000E+01, 5.10000E+01, 5.92000E+01, 5.40000E+01, 5.50000E+01, 5.00000E+01, 6.10000E+01, 5.20000E+01, 6.60000E+01, 5.14000E+01, 5.52000E+01, 5.06000E+01), .Dim=c(2, 19)), mean.status= structure(.Data= c(3.00000E+00, 2.00000E+00, 3.00000E+00, 3.15000E+00, 3.10000E+00, 3.11111E+00, 2.73333E+00, 2.75194E+00, 3.02941E+00, 3.27273E+00, 3.00000E+00, 3.05797E+00, 2.69355E+00, 3.27500E+00, 2.55714E+00, 2.60000E+00, 3.20000E+00, 2.80000E+00, 2.60000E+00, 3.00000E+00, 2.00000E+00, 3.00000E+00, 3.15000E+00, 3.10000E+00, 3.11111E+00, 2.54762E+00, 2.80451E+00, 3.03030E+00, 3.22727E+00, 3.00000E+00, 3.09722E+00, 2.65000E+00, 3.24390E+00, 2.61972E+00, 2.60000E+00, 2.80000E+00, 3.20000E+00, 3.20000E+00), .Dim=c(2, 19)), mean.walk= structure(.Data= c(3.87000E+02, 3.92000E+02, 3.39000E+02, 3.46000E+02, 2.64720E+02, 3.46000E+02, 3.48500E+02, 3.41000E+02, 3.40000E+02, 3.23620E+02, 3.55000E+02, 3.44000E+02, 3.21000E+02, 2.72000E+02, 3.44000E+02, 3.39600E+02, 3.36600E+02, 3.92000E+02, 3.40800E+02, 3.87000E+02, 3.92000E+02, 3.39000E+02, 3.46000E+02, 2.64720E+02, 3.46000E+02, 3.60900E+02, 3.48900E+02, 3.31000E+02, 3.23040E+02, 3.60000E+02, 3.30000E+02, 3.37000E+02, 3.16000E+02, 3.39000E+02, 3.92400E+02, 3.51200E+02, 3.17600E+02, 3.74600E+02), .Dim=c(2, 19)), mean.pvr= structure(.Data= c(9.57744E+02, 7.76000E+02, 7.44000E+02, 1.14700E+03, 9.28000E+02, 1.54900E+03, 8.63300E+02, 7.54900E+02, 7.83000E+02, 1.42600E+03, 9.42000E+02, 8.80000E+02, 8.80000E+02, 1.28000E+03, 1.05100E+03, 1.23760E+03, 1.06840E+03, 1.05680E+03, 8.77000E+02, 9.57744E+02, 7.76000E+02, 7.44000E+02, 1.14700E+03, 9.28000E+02, 1.54900E+03, 8.63300E+02, 8.56800E+02, 8.15000E+02, 1.51100E+03, 8.96000E+02, 1.01400E+03, 8.80000E+02, 1.28000E+03, 9.18000E+02, 1.14680E+03, 1.26360E+03, 7.69000E+02, 1.21300E+03), .Dim=c(2, 19)), vague.prec=1.00000E-06, mean.vague.prec=1.00000E-06, upper.lsd=5.00000E+03, N.beta=1.10000E+01, study.beta= structure(.Data= c(0.00000E+00, 1.00000E+00, 0.00000E+00, 1.00000E+00, 0.00000E+00, 1.00000E+00, 0.00000E+00, 0.00000E+00, 0.00000E+00, 1.00000E+00, 1.00000E+00, 1.00000E+00, 1.00000E+00, 0.00000E+00, 0.00000E+00, 0.00000E+00, 0.00000E+00, 0.00000E+00, 0.00000E+00, 0.00000E+00, -1.00000E+00, 0.00000E+00, -1.00000E+00, 0.00000E+00, 0.00000E+00, 0.00000E+00, 0.00000E+00, 0.00000E+00, 0.00000E+00, 0.00000E+00, 0.00000E+00, 0.00000E+00, 1.00000E+00, 0.00000E+00, 0.00000E+00, 0.00000E+00, 0.00000E+00, 0.00000E+00, 0.00000E+00, 1.00000E+00, 1.00000E+00, 1.00000E+00, 0.00000E+00, 0.00000E+00, 0.00000E+00, 0.00000E+00, 1.00000E+00, 1.00000E+00, 0.00000E+00, 0.00000E+00, 0.00000E+00, 0.00000E+00, 0.00000E+00, 0.00000E+00, 0.00000E+00, 0.00000E+00, 0.00000E+00, 0.00000E+00, 0.00000E+00, 0.00000E+00, 0.00000E+00, 1.00000E+00, 0.00000E+00, 1.00000E+00, 0.00000E+00, 0.00000E+00, 0.00000E+00, 0.00000E+00, 0.00000E+00, 0.00000E+00, 0.00000E+00, 0.00000E+00, 0.00000E+00, 0.00000E+00, 0.00000E+00, 0.00000E+00, 0.00000E+00, 0.00000E+00, 0.00000E+00, 0.00000E+00, 0.00000E+00, 0.00000E+00, 0.00000E+00, 1.00000E+00, 0.00000E+00, 0.00000E+00, 0.00000E+00, 0.00000E+00, 0.00000E+00, 0.00000E+00, 0.00000E+00, 0.00000E+00, 0.00000E+00, 0.00000E+00, 0.00000E+00, 1.00000E+00, 0.00000E+00, 0.00000E+00, 0.00000E+00, 0.00000E+00, 0.00000E+00, 0.00000E+00, 0.00000E+00, 0.00000E+00, 0.00000E+00, 0.00000E+00, 0.00000E+00, 0.00000E+00, 0.00000E+00, 0.00000E+00, 0.00000E+00, 0.00000E+00, 0.00000E+00, 0.00000E+00, 0.00000E+00, 0.00000E+00, 0.00000E+00, 0.00000E+00, 0.00000E+00, 0.00000E+00, 0.00000E+00, 0.00000E+00, 0.00000E+00, 0.00000E+00, 0.00000E+00, 0.00000E+00, 0.00000E+00, 0.00000E+00, 1.00000E+00, 0.00000E+00, 0.00000E+00, 0.00000E+00, 0.00000E+00, 0.00000E+00, 0.00000E+00, 0.00000E+00, 0.00000E+00, 0.00000E+00, 0.00000E+00, 0.00000E+00, 0.00000E+00, 0.00000E+00, 0.00000E+00, 0.00000E+00, 0.00000E+00, 0.00000E+00, 0.00000E+00, 0.00000E+00, 1.00000E+00, 0.00000E+00, 0.00000E+00, 0.00000E+00, 0.00000E+00, 0.00000E+00, 0.00000E+00, 0.00000E+00, 0.00000E+00, 0.00000E+00, 0.00000E+00, 0.00000E+00, 0.00000E+00, 0.00000E+00, 0.00000E+00, 0.00000E+00, 0.00000E+00, 0.00000E+00, 0.00000E+00, 0.00000E+00, 1.00000E+00, 0.00000E+00, 0.00000E+00, 0.00000E+00, 0.00000E+00, 0.00000E+00, 0.00000E+00, 0.00000E+00, 0.00000E+00, 0.00000E+00, 0.00000E+00, 0.00000E+00, 0.00000E+00, 0.00000E+00, 0.00000E+00, 0.00000E+00, 0.00000E+00, 0.00000E+00, 0.00000E+00, 0.00000E+00, 1.00000E+00, 0.00000E+00, 0.00000E+00, 0.00000E+00, 0.00000E+00, 0.00000E+00, 0.00000E+00, 0.00000E+00, 0.00000E+00, 0.00000E+00, 0.00000E+00, 0.00000E+00, 0.00000E+00, 0.00000E+00, 0.00000E+00, 0.00000E+00, 0.00000E+00, 0.00000E+00, 0.00000E+00, 0.00000E+00, 1.00000E+00), .Dim=c(11, 19)), study.beta.control= structure(.Data= c(0.00000E+00, 0.00000E+00, 0.00000E+00, 0.00000E+00, 0.00000E+00, 0.00000E+00, 0.00000E+00, 0.00000E+00, 0.00000E+00, 0.00000E+00, 0.00000E+00, 0.00000E+00, 0.00000E+00, 0.00000E+00, 0.00000E+00, 0.00000E+00, 0.00000E+00, 0.00000E+00, 0.00000E+00, 0.00000E+00, 0.00000E+00, 0.00000E+00, 0.00000E+00, 0.00000E+00, 0.00000E+00, 0.00000E+00, 0.00000E+00, 0.00000E+00, 1.00000E+00, 0.00000E+00, 0.00000E+00, 0.00000E+00, 0.00000E+00, 0.00000E+00, 0.00000E+00, 0.00000E+00, 0.00000E+00, 0.00000E+00, 0.00000E+00, 0.00000E+00, 0.00000E+00, 0.00000E+00, 0.00000E+00, 0.00000E+00, 0.00000E+00, 0.00000E+00, 0.00000E+00, 0.00000E+00, 0.00000E+00, 0.00000E+00, 0.00000E+00, 0.00000E+00, 0.00000E+00, 0.00000E+00, 0.00000E+00, 0.00000E+00, 0.00000E+00, 0.00000E+00, 0.00000E+00, 0.00000E+00, 0.00000E+00, 0.00000E+00, 0.00000E+00, 0.00000E+00, 0.00000E+00, 0.00000E+00, 0.00000E+00, 0.00000E+00, 0.00000E+00, 0.00000E+00, 0.00000E+00, 0.00000E+00, 0.00000E+00, 0.00000E+00, 0.00000E+00, 0.00000E+00, 0.00000E+00, 0.00000E+00, 0.00000E+00, 0.00000E+00, 0.00000E+00, 0.00000E+00, 0.00000E+00, 0.00000E+00, 0.00000E+00, 0.00000E+00, 0.00000E+00, 0.00000E+00, 0.00000E+00, 0.00000E+00, 0.00000E+00, 0.00000E+00, 0.00000E+00, 0.00000E+00, 0.00000E+00, 0.00000E+00, 0.00000E+00, 0.00000E+00, 0.00000E+00, 0.00000E+00, 0.00000E+00, 0.00000E+00, 0.00000E+00, 0.00000E+00, 0.00000E+00, 0.00000E+00, 0.00000E+00, 0.00000E+00, 0.00000E+00, 0.00000E+00, 0.00000E+00, 0.00000E+00, 0.00000E+00, 0.00000E+00, 0.00000E+00, 0.00000E+00, 0.00000E+00, 0.00000E+00, 0.00000E+00, 0.00000E+00, 0.00000E+00, 0.00000E+00, 0.00000E+00, 0.00000E+00, 0.00000E+00, 0.00000E+00, 0.00000E+00, 0.00000E+00, 0.00000E+00, 0.00000E+00, 0.00000E+00, 0.00000E+00, 0.00000E+00, 0.00000E+00, 0.00000E+00, 0.00000E+00, 0.00000E+00, 0.00000E+00, 0.00000E+00, 0.00000E+00, 0.00000E+00, 0.00000E+00, 0.00000E+00, 0.00000E+00, 0.00000E+00, 0.00000E+00, 0.00000E+00, 0.00000E+00, 0.00000E+00, 0.00000E+00, 0.00000E+00, 0.00000E+00, 0.00000E+00, 0.00000E+00, 0.00000E+00, 0.00000E+00, 0.00000E+00, 0.00000E+00, 0.00000E+00, 0.00000E+00, 0.00000E+00, 0.00000E+00, 0.00000E+00, 0.00000E+00, 0.00000E+00, 0.00000E+00, 0.00000E+00, 0.00000E+00, 0.00000E+00, 0.00000E+00, 0.00000E+00, 0.00000E+00, 0.00000E+00, 0.00000E+00, 0.00000E+00, 0.00000E+00, 0.00000E+00, 0.00000E+00, 0.00000E+00, 0.00000E+00, 0.00000E+00, 0.00000E+00, 0.00000E+00, 0.00000E+00, 0.00000E+00, 0.00000E+00, 0.00000E+00, 0.00000E+00, 0.00000E+00, 0.00000E+00, 0.00000E+00, 0.00000E+00, 0.00000E+00, 0.00000E+00, 0.00000E+00, 0.00000E+00, 0.00000E+00, 0.00000E+00, 0.00000E+00, 0.00000E+00, 0.00000E+00, 0.00000E+00, 0.00000E+00, 0.00000E+00, 0.00000E+00, 0.00000E+00, 0.00000E+00, 0.00000E+00, 0.00000E+00), .Dim=c(11, 19)), within.cov=c(1.00000E+00, 0.00000E+00, 1.00000E+00, 0.00000E+00, 1.00000E+00, 0.00000E+00), across.cov=c(0.00000E+00, 0.00000E+00, 0.00000E+00, 0.00000E+00, 0.00000E+00, 0.00000E+00), inter.cov=c(0.00000E+00, 0.00000E+00, 0.00000E+00, 0.00000E+00, 0.00000E+00, 0.00000E+00), observational=c(1.00000E+00, 1.00000E+00, 1.00000E+00, 1.00000E+00, 1.00000E+00, 1.00000E+00, 0.00000E+00, 0.00000E+00, 0.00000E+00, 0.00000E+00, 0.00000E+00, 0.00000E+00, 0.00000E+00, 0.00000E+00, 0.00000E+00, 0.00000E+00, 0.00000E+00, 0.00000E+00, 0.00000E+00), deltaA=c(1.00000E+00, 1.00000E+00, 1.00000E+00, 1.00000E+00, 1.00000E+00, 1.00000E+00, 1.00000E+00, 1.00000E+00, 1.00000E+00, 1.00000E+00, 1.00000E+00, 1.00000E+00, 1.00000E+00, 1.00000E+00, 1.00000E+00, 1.00000E+00, 1.00000E+00, 1.00000E+00, 1.00000E+00), deltaC=c(1.00000E+00, 1.00000E+00, 1.00000E+00, 1.00000E+00, 1.00000E+00, 1.00000E+00, 1.00000E+00, 1.00000E+00, 1.00000E+00, 1.00000E+00, 1.00000E+00, 1.00000E+00, 1.00000E+00, 1.00000E+00, 1.00000E+00, 1.00000E+00, 1.00000E+00, 1.00000E+00, 1.00000E+00), random.effects=1.00000E+00)*

*#################################################################################################################*
