# Supplementary material for: Network meta-analysis combining individual patient and aggregate data from a mixture of study designs with an application to pulmonary arterial hypertension
Source: BMC Med Res Methodol. 2015 Apr 12;15:34. doi: 10.1186/s12874-015-0007-0 (PMC4403724; doi:10.1186/s12874-015-0007-0)
Supplement: Additional file 4: — Further parameter estimates from NMA Model M3: Covariate adjusted NMA of IPD and aggregate data. [file 12874_2015_7_MOESM4_ESM.docx]

**Additional file 4. Further parameter estimates from NMA Model M3: Covariate adjusted NMA of IPD and aggregate data**

###### Table A.1 provides a more complete summary, with means, standard deviations, 95% credible intervals and the medians, of the posterior distributions of the parameters of model M3 of Sections 2.3 and 3.2. Among these reported parameters are the short term placebo improvements in 6MWD for each of the included studies are the $\alpha_{i}$s, and their variation illustrates the flexibility provided by random effects. The mean of the posterior distribution of the standard deviation of these placebo improvements, $\sigma_{\alpha}$, and of the total treatment effect, $\sigma_{\beta}$, and total change in 6MWD from baseline, $\sigma$, are comfortably within the range of the specified prior distribution (a Uniform with limits at 0 and 50). This indicates that our model had the desirable property of being insensitive to the prior – a property further verified by prior sensitivity analysis.

###### Table A.1 Parameter estimates from NMA model M3 with no across-study covariate adjustments on the main effect, within-study adjustments for AGE, STATUS and PVR on the main effect and no covariate adjustments on the treatment effect of imatinib. Based on 250000 simulations following a burn-in of 100000. DIC=1870.50.

|  | **Mean** | **SD** | **2.5%** | **50%** | **97.5%** |
| --- | --- | --- | --- | --- | --- |
| $\sigma$ (standard deviation of total short term change in 6MWD in each trial) | 48.72 | 1.19 | 45.59 | 49.06 | 49.96 |
| $\alpha_{i}$, Jacobs 2009 | 4.39 | 11.31 | -18.77 | 4.06 | 28.59 |
| $\alpha_{i}$, Akagi 2008 | -0.40 | 10.70 | -25.33 | 0.79 | 18.62 |
| $\alpha_{i}$, Channick 2006 | 5.74 | 10.99 | -15.13 | 4.91 | 30.59 |
| $\alpha_{i}$, Hoeper 2003 | 9.82 | 10.65 | -7.45 | 8.12 | 34.89 |
| $\alpha_{i}$, Mathai 2007 | 3.30 | 10.36 | -18.48 | 3.33 | 24.90 |
| $\alpha_{i}$, Hoeper 2004 | 5.39 | 10.63 | -15.20 | 4.72 | 29.03 |
| $\alpha_{i}$, Barst 2011 | 9.54 | 8.45 | -4.90 | 8.63 | 28.04 |
| $\alpha_{i}$, Simonneau 2008 | 2.85 | 6.85 | -10.84 | 2.82 | 16.99 |
| $\alpha_{i}$, McLaughlin 2006 | 2.99 | 7.58 | -12.38 | 2.97 | 18.58 |
| $\alpha_{i}$, Humbert 2004 | 5.24 | 9.58 | -13.71 | 4.78 | 25.99 |
| $\alpha_{i}$, Badesch 2002 | 7.36 | 10.21 | -10.89 | 6.20 | 30.75 |
| $\alpha_{i}$, Rubin 2002 | 0.57 | 7.31 | -14.92 | 0.96 | 14.39 |
| $\alpha_{i}$, Barst 2006 | -2.07 | 7.91 | -19.02 | -1.44 | 12.19 |
| $\alpha_{i}$, Barst 1996 | 2.59 | 9.72 | -18.52 | 2.91 | 21.80 |
| $\alpha_{i}$, Galie 2005 | 3.28 | 6.81 | -10.31 | 3.20 | 17.33 |
| $\alpha_{i}$, (imatinib to E+P5) | 3.50 | 6.97 | -10.59 | 3.50 | 17.59 |
| $\alpha_{i}$, (imatinib to E+Pr) | 4.97 | 8.80 | -12.30 | 4.56 | 23.97 |
| $\alpha_{i}$, (imatinib to E+P5+Pr) | -1.40 | 6.89 | -16.28 | -0.84 | 10.89 |
| $\alpha_{i}$, (imatinib to Pr+P5) | 14.29 | 10.41 | -2.09 | 12.91 | 37.38 |
| $\alpha$ (mean of $\alpha_{i}$s) | 4.30 | 4.77 | -4.73 | 4.16 | 14.17 |
| $\sigma_{\alpha}$ (standard deviation of $\alpha_{i}$s) | 8.73 | 5.32 | 0.52 | 8.24 | 20.65 |
| $\beta_{1}$ (E to placebo) | 35.71 | 9.00 | 18.15 | 35.57 | 54.07 |
| $\beta_{2}$ (Pr to placebo) | 31.04 | 13.88 | 4.87 | 30.53 | 59.61 |
| $\beta_{3}$ (Pr to E) | 31.95 | 11.77 | 7.80 | 32.35 | 54.60 |
| $\beta_{4}$ (P5 to E) | 28.18 | 13.91 | 0.32 | 28.38 | 55.25 |
| $\beta_{5}$ (P5 to Pr) | 26.85 | 12.83 | 0.21 | 27.03 | 52.49 |
| $\beta_{6}$ (Pr to E+P5) | 34.65 | 40.07 | -42.18 | 34.73 | 113.20 |
| $\beta_{7}$ (P5 to placebo) | 46.66 | 14.76 | 16.64 | 46.77 | 75.37 |
| $\beta_{8}$ (QTI to E+P5) | 40.06 | 13.85 | 13.38 | 40.06 | 67.67 |
| $\beta_{9}$ (QTI to E+Pr) | 43.10 | 18.76 | 5.71 | 43.04 | 79.68 |
| $\beta_{10}$ (QTI to Pr+P5) | 34.49 | 12.24 | 11.05 | 34.10 | 59.17 |
| $\beta_{11}$ (QTI to E+P5+Pr) | 25.50 | 19.60 | -13.59 | 25.93 | 63.15 |
| $\sigma_{\beta}$ | 7.91 | 4.76 | 0.39 | 7.63 | 17.63 |
| $\pi_{1}$ (AGE) | -0.90 | 0.32 | -1.52 | -0.90 | -0.27 |
| $\pi_{3}$ (STATUS) | -11.89 | 8.33 | -28.20 | -11.87 | 4.44 |
| $\pi_{5}$ (PVR) | -0.05 | 0.01 | -0.07 | -0.05 | -0.02 |
| imatinib v Pr to E+P5 | 5.41 | 41.74 | -76.65 | 5.49 | 85.24 |
| Deviance | 1834.56 | 15.89 | 1799.00 | 1837.00 | 1860.00 |
